# Acute kidney injury in critical COVID-19: a multicenter cohort analysis in seven large hospitals in Belgium

**DOI:** 10.1186/s13054-022-04086-x

**Published:** 2022-07-25

**Authors:** Hannah Schaubroeck, Wim Vandenberghe, Willem Boer, Eva Boonen, Bram Dewulf, Camille Bourgeois, Jasperina Dubois, Alexander Dumoulin, Tom Fivez, Jan Gunst, Greet Hermans, Piet Lormans, Philippe Meersseman, Dieter Mesotten, Björn Stessel, Marc Vanhoof, Greet De Vlieger, Eric Hoste

**Affiliations:** 1 Department of Intensive Care Medicine, Department of Internal Medicine and Pediatrics, Ghent University Hospital, Ghent University, Corneel Heymanslaan 10, 9000 Ghent, Belgium; 2 Department of Anesthesiology, Intensive Care Medicine, Emergency Medicine and Pain Medicine, Ziekenhuis Oost Limburg ZOL, Genk, Belgium; 3 Department of Intensive Care Medicine, AZ Turnhout, Turnhout, Belgium; 4 Department of Anaesthesiology and Critical Care Medicine, AZ Sint-Jan Brugge-Oostende AV, Brugge, Belgium; 5 Department of Intensive Care Medicine and Anaesthesiology, Jessa Hospital, Hasselt, Belgium; 6 Department of Intensive Care Medicine, AZ Delta, Roeselare, Belgium; 7 Department of Intensive Care Medicine, University Hospitals Leuven, Leuven, Belgium; 8 Laboratory of Intensive Care Medicine, Department of Cellular and Molecular Medicine, KU Leuven, Leuven, Belgium; 9 Department of General Internal Medicine, Medical Intensive Care Unit, University Hospitals Leuven, Leuven, Belgium; 10 Faculty of Medicine and Life Sciences, UHasselt, LCRC, Diepenbeek, Belgium; 11 Research Foundation-Flanders (FWO), Brussels, Belgium

**Keywords:** Acute kidney injury, Kidney replacement therapy, Renal replacement therapy, COVID-19, Intensive care unit, Epidemiology, Mortality, KDIGO, Urine output, Serum creatinine

## Abstract

**Background:**

Acute kidney injury (AKI) has been reported as a frequent complication of critical COVID-19. We aimed to evaluate the occurrence of AKI and use of kidney replacement therapy (KRT) in critical COVID-19, to assess patient and kidney outcomes and risk factors for AKI and differences in outcome when the diagnosis of AKI is based on urine output (UO) or on serum creatinine (sCr).

**Methods:**

Multicenter, retrospective cohort analysis of patients with critical COVID-19 in seven large hospitals in Belgium. AKI was defined according to KDIGO within 21 days after ICU admission. Multivariable logistic regression analysis was used to explore the risk factors for developing AKI and to assess the association between AKI and ICU mortality.

**Results:**

Of 1286 patients, 85.1% had AKI, and KRT was used in 9.8%. Older age, obesity, a higher APACHE II score and use of mechanical ventilation at day 1 of ICU stay were associated with an increased risk for AKI. After multivariable adjustment, all AKI stages were associated with ICU mortality. AKI was based on sCr in 40.1% and UO in 81.5% of patients. All AKI stages based on sCr and AKI stage 3 based on UO were associated with ICU mortality. Persistent AKI was present in 88.6% and acute kidney disease (AKD) in 87.6%. Rapid reversal of AKI yielded a better prognosis compared to persistent AKI and AKD. Kidney recovery was observed in 47.4% of surviving AKI patients.

**Conclusions:**

Over 80% of critically ill COVID-19 patients had AKI. This was driven by the high occurrence rate of AKI defined by UO criteria. All AKI stages were associated with mortality (NCT04997915).

**Supplementary Information:**

The online version contains supplementary material available at 10.1186/s13054-022-04086-x.

## Background

In December 2019, severe acute respiratory syndrome coronavirus 2 (SARS-CoV-2) appeared for the first time in China, causing an ongoing pandemic of coronavirus disease 2019 (COVID-19). COVID-19 overwhelmed health care systems with an immense strain on intensive care units (ICU) and caused excess mortality of almost 15 million deaths worldwide [1, 2]. Acute kidney injury (AKI) has been reported as a complication of critical COVID-19 in 25–76%, and the use of kidney replacement therapy (KRT) in 5–44% [3–6] (Additional file 1: Table S1: Overview of studies reporting on AKI in critical COVID-19 patients). The Acute Disease Quality Initiative (ADQI) formulated in its 25th conference the need for more detailed epidemiological data [7].

The objective of this study was to evaluate the occurrence of AKI and use of KRT in critical COVID-19 in several large hospitals in Belgium and its association with mortality. We aimed to evaluate the association of baseline risk factors and therapeutic strategies in critical COVID-19 with AKI and with patient outcomes and to explore the difference in outcome when the diagnosis of AKI is based on urine output (UO) (AKI-UO) or on serum creatinine (sCr) (AKI-sCr) alone according to the 2012 KDIGO guidelines. Finally, we aimed to assess kidney outcomes, especially rapid reversal of AKI, kidney recovery and occurrence of acute kidney disease (AKD).

## Methods

### Study design, setting and participants

We conducted a multicenter, retrospective cohort analysis in adult (≥ 18 y) patients with critical COVID-19 admitted to several ICU departments (medical, surgical or mixed) in seven large hospitals in Flanders between February 1, 2020, and January 31, 2021. In some of the participating hospitals, ICU capacity was increased by creating extra ICU units outside the regular ICU department, e.g., in the post-anesthesia care unit.

We included patients if SARS-CoV-2 infection was confirmed by real‐time reverse‐transcriptase polymerase chain reaction (PCR) on nasopharyngeal or oropharyngeal swabs, bronchial aspirate or broncho-alveolar lavage fluid. Patients were excluded when there was a diagnosis of COVID-19 based on clinical symptoms or chest CT scan without confirmation by PCR, when patients who had a positive SARS-CoV-2 PCR were admitted to ICU for other medical reasons and when patients had end-stage kidney disease on chronic KRT.

The study was registered on clinicaltrials.gov (NCT04997915). The STROBE guidelines for cohort studies were applied (Additional file 1: Table S2).

### Data collection and management

We collected patients’ baseline characteristics, comorbidities, medication at home, specific treatment for COVID-19, potentially nephrotoxic drugs, biochemical parameters, use of mechanical ventilation, PaO_2_/FiO_2_ ratio, use of veno-venous extracorporeal membrane oxygenation (VV-ECMO), use of vasoactive drugs, severity of illness and kidney and patient outcomes. All data were extracted from the electronic patient data management systems, pseudonymized and collected by each participating center. Pseudonymized data from all centers were merged into one large database, remote monitoring was applied to check validity of delivered data, and extensive data cleaning was performed.

### Definitions

We described all relevant definitions (Additional file 1: Definitions Used, Table S3, Fig. S1). AKI, rapid reversal AKI, persistent AKI, AKD and kidney recovery were defined according to KDIGO and ADQI guidelines [8–10].

### Outcomes

The pre-specified primary outcome was the rate of COVID-19-associated AKI and its stages assessed up to day 21 of ICU admission. We explored the association between AKI and ICU and hospital mortality. We also reported the association between AKI and 30-day mortality in patients who had at least 30 days of follow-up. We assessed the risk factors for developing AKI and for ICU mortality. Secondary outcomes included patient and kidney outcomes of AKI defined by serum creatinine (sCr) criteria (AKI-sCr) and urine output (UO) criteria (AKI-UO) separately. We also evaluated the rates and outcomes of rapid reversal AKI, persistent AKI, and AKD. Additionally, we assessed kidney recovery, persistent use of KRT and ICU and hospital length of stay (LOS).

### Statistical analysis

Epidemiological data were reported as number (proportion) and median (25% quartile, 75% quartile). Univariate analysis of continuous variables was performed with the Mann–Whitney U test and Wilcoxon signed-rank test when appropriate. Discrete variables were evaluated with Fisher’s exact test or chi-square test. Multivariable logistic regression analysis was used to explore risk factors for development of AKI and for assessment of the association between AKI and ICU mortality. Variables selected for inclusion in the models were those with a biological or plausible rationale and a *p* value of 0.25 or less in bivariate analysis of patients with and without AKI as well as ICU survivors and non-survivors. We used backward selection (Wald) to evaluate variables for inclusion and checked for colinearity and interaction. The Hosmer–Lemeshow test and the area under the receiver operating characteristic curve of the predicted probability of a model were used to assess goodness-of-fit and discrimination of the models (AUC-ROC, c statistic). Missing values were considered missing at random and were not replaced. All statistical analyses were performed with SPSS Statistics 27 (IBM Corporation and Others^®^). Figures were made with SigmaPlot (Version 14.5, Systat Software Inc.). Confidence intervals of proportions were calculated according to Gardner et al. [11]. A sensitivity analysis was performed on the cohort with true baseline sCr, i.e., recorded within 7–365 days before ICU admission.

### Ethical committee

This study was approved by the Ethics Committee (EC) of the participating hospitals. The EC of the Ghent University Hospital acted as central EC (BC-08285). Informed consent of participants was waived. This study was conducted in accordance with the ethical and scientific principles of Good Clinical Practice.

## Results

### Patient characteristics

**Fig. 1:**
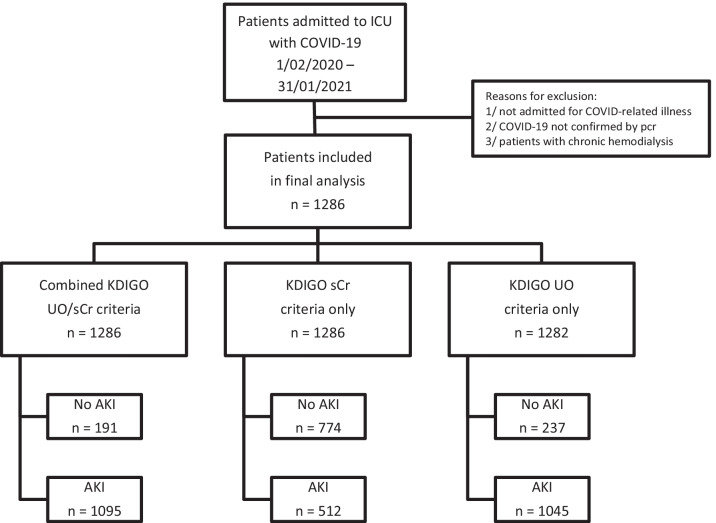
Study flowchart. Flowchart summarizing patient selection and inclusion process as well as number of patients with AKI according to the full KDIGO definition, to sCr or to UO criteria

In the final analysis, 1286 patients were included (Fig. 1). Baseline demographics, severity-of-illness parameters, and treatment during ICU admission are outlined in Table 1. True baseline sCr was available in 69.1% of patients (95% CI 66.6%, 71.7%) (Additional file 1: Table S4).

**Table 1:** (a) Baseline demographics, (b) severity-of-illness parameters, and (c) treatment during ICU admission

	All *n* = 1286	No AKI *n* = 191 14.9%	AKI *n* = 1095 85.1%	*p* Value
*(a) Baseline demographics*				
Sex (M/F) (*n* = 1286)	879 (68.4%)/407 (31.6%)	135 (70.7%)/56 (29.3%)	744 (67.9%)/351 (32.1%)	0.453
Age (y) (*n* = 1285)	69 (59, 77)	63 (53, 73)	69 (60, 77)	< 0.001
Body mass index (kg/m^2^) (*n* = 1262)	27.8 (25.0, 32.0)	26.9 (24.4, 30.1)	28.1 (25.2, 32.5)	< 0.001
Baseline sCr (mg/dL) (*n* = 1286)	0.85 (0.67, 1.07)	0.84 (0.70, 1.03)	0.85 (0.67, 1.08)	0.781
eGFR (mL/min/1.73m^2^) (*n* = 1285)	84 (67, 98)	86 (74, 101)	83 (66, 97)	0.014
CKD (eGFR < 60) (*n* = 1285)	252 (19.6%)	28 (14.7%)	224 (20.5%)	0.062
COVID day at Hospital admission (*n* = 849)	6.0 (3.0, 9.0)	7.0 (3.0, 10.0)	6.0 (3.0, 8.0)	0.295
COVID day at ICU admission (*n* = 850)	8.0 (5.0, 11.6)	9.0 (4.5, 11.6)	8.0 (5.0, 11.6)	0.732
*Comorbidities*				
Arterial hypertension (*n* = 1042)	492 (47.2%)	60 (39.7%)	432 (48.5%)	0.046
Cardiovascular disease (*n* = 948)	391 (41.2%)	51 (36.7%)	340 (42.0%)	0.238
COPD/asthma (*n* = 949)	145 (15.3%)	19 (13.7%)	126 (15.6%)	0.568
Diabetes (*n* = 955)	245 (25.7%)	23 (16.8%)	222 (27.1%)	0.010
Cirrhosis (*n* = 948)	18 (1.9%)	2 (1.4%)	16 (2.0%)	1.000
Cancer (*n* = 947)	112 (11.8%)	12 (8.7%)	100 (12.4%)	0.218
Immunocompromised (*n* = 948)	95 (10.0%)	18 (12.9%)	77 (9.5%)	0.213
*Medication at home*				
ACEI/ARBs (*n* = 727)	242 (33.3%)	28 (26.2%)	214 (34.5%)	0.091
Immunosuppressive drugs (*n* = 947)	56 (5.9%)	11 (8.0%)	45 (5.6%)	0.268
*Severity of illness at first day of ICU admission* *(In case of hospital transfer, first day of ICU in investigating center)*				
*(b) Severity-of-illness parameters*				
APACHE II (*n* = 883)	16.0 (13.0, 22.0)	14.0 (10.3, 17.0)	17.0 (13.0, 22.0)	< 0.001
SAPS II (*n* = 855)	39 (31, 48)	31 (24, 41)	40 (32, 49)	< 0.001
SOFA (*n* = 926)	5 (3, 10)	3 (2, 5)	5 (3, 10)	< 0.001
Lymphocytopenia (< 1000/µL) (*n* = 813)	518 (63.7%)	85 (72.6%)	433 (62.2%)	0.030
Ferritin (µg/L) (*n* = 636)	1226 (617, 1942)	1390 (612, 2486)	1200 (617, 1916)	0.299
CRP (mg/L) (*n* = 1066)	132.5 (74.2, 209.8)	97.1 (54.5, 190.0)	138.3 (79.1, 213.1)	< 0.001
D-dimers (ng/mL) (*n* = 783)	1160 (573, 2344)	750 (290, 1623)	1246 (600, 2520)	< 0.001
PaO_2_/FiO_2_ (*n* = 970)	89 (68, 127)	114 (75, 160)	88 (67, 124)	< 0.001
HFO (*n* = 923)	512 (55.5%)	55 (50.5%)	457 (56.1%)	0.262
NIV (*n* = 952)	91 (9.6%)	7 (5.1%)	84 (10.3%)	0.436
IMV (*n* = 962)	351 (36.5%)	16 (14.7%)	335 (39.3%)	< 0.001
Prone (*n* = 847)	63 (7.4%)	6 (5.7%)	57 (7.7%)	0.472
VV-ECMO (*n* = 838)	32 (3.8%)	0 (0%)	32 (4.4%)	0.025
Vasoactive drugs (*n* = 1286)	387 (30.1%)	32 (16.8%)	355 (32.4%)	< 0.001
MAP (mmHg) (lowest) (*n* = 971)	71 (61, 86)	75 (65, 87)	71 (61, 86)	0.175
*Severity of illness @ first day of AKI*				
AKI stage				
Stage 1	472 (45.1%)			
Stage 2	522 (49.9%)			
Stage 3	52 (5.0%)			
SOFA score (*n* = 353)	9 (5, 11)			
PaO_2_/FiO_2_ (*n* = 1044)	98 (69, 137)			
HFO (*n* = 573)	290 (50.6%)			
NIV (*n* = 659)	49 (7.4%)			
IMV (*n* = 836)	400 (47.8%)			
VV-ECMO (*n* = 608)	42 (6.9%)			
Prone position (*n* = 638)	118 (18.5%; 29.2% of patients on IMV)			
Vasoactive drugs (*n* = 503)	322 (64.0%)			
MAP (mmHg) (lowest) (*n* = 854)	64 (55, 76)			
Lymphocytopenia (< 1000/µL) (*n* = 781)	658 (84.3%)			
Ferritin (µg/L) (*n* = 713)	1043 (414, 1700)			
CRP (mg/L) (*n* = 1060)	123 (63, 210)			
D-dimers (*n* = 709)	950 (340, 2121)			
*(c) Treatment during ICU admission*				
Corticosteroids (*n* = 1043)	780 (74.8%)	96 (63.6%)	684 (76.7%)	< 0.001
Hydroxychloroquine (*n* = 1285)	333 (25.9%)	40 (20.9%)	293 (26.8%)	0.089
Remdesivir (*n* = 1285)	193 (15.0%)	39 (20.4%)	154 (14.1%)	0.024
Anti-IL1 or anti-IL6 (*n* = 1286)	54 (4.2%)	5 (2.6%)	49 (4.5%)	0.238
Convalescent COVID-19 plasma (*n* = 1214)	15 (1.2%)	2 (1.1%)	13 (1.3%)	1.000
NSAIDs (*n* = 1285)	48 (3.7%)	5 (2.6%)	43 (3.9%)	0.377
Aminoglycoside (*n* = 1041)	83 (8.0%)	3 (2.0%)	80 (9.0%)	0.003
Vancomycin (*n* = 1285)	215 (16.7%)	5 (2.6%)	210 (19.2%)	< 0.001
Vasoactive drugs (*n* = 1286)	648 (50.4%)	37 (19.4%)	611 (55.8%)	< 0.001
IMV (*n* = 870)	560 (64.4%)	18 (23.1%)	542 (68.4%)	< 0.001

*AKI* acute kidney injury, *M* male, *F* female, *y* years, *sCr* serum creatinine, *eGFR* estimated glomerular filtration rate, *COVID* coronavirus disease, *ICU* intensive care unit, *COPD* chronic obstructive pulmonary disease, *ACEI* angiotensin-converting enzyme inhibitors, *ARBs* angiotensin receptor blockers, *CRP* C-reactive protein, *PaO2* arterial oxygen pressure, *FiO2* fraction of inspired oxygen, *HFO* high-flow oxygen, *NIV* noninvasive ventilation, *IMV* invasive mechanical ventilation, *vv-ECMO* veno-venous extracorporeal membrane oxygenation, *MAP* mean arterial pressure, *IL* interleukin, *NSAIDs* nonsteroidal anti-inflammatory drugs

Continuous variables are reported as median and interquartile ranges; categorical variables are reported as absolute numbers and frequencies

### Primary outcomes

#### AKI according to full KDIGO definition

##### Occurrence and stages

**Fig. 2:**
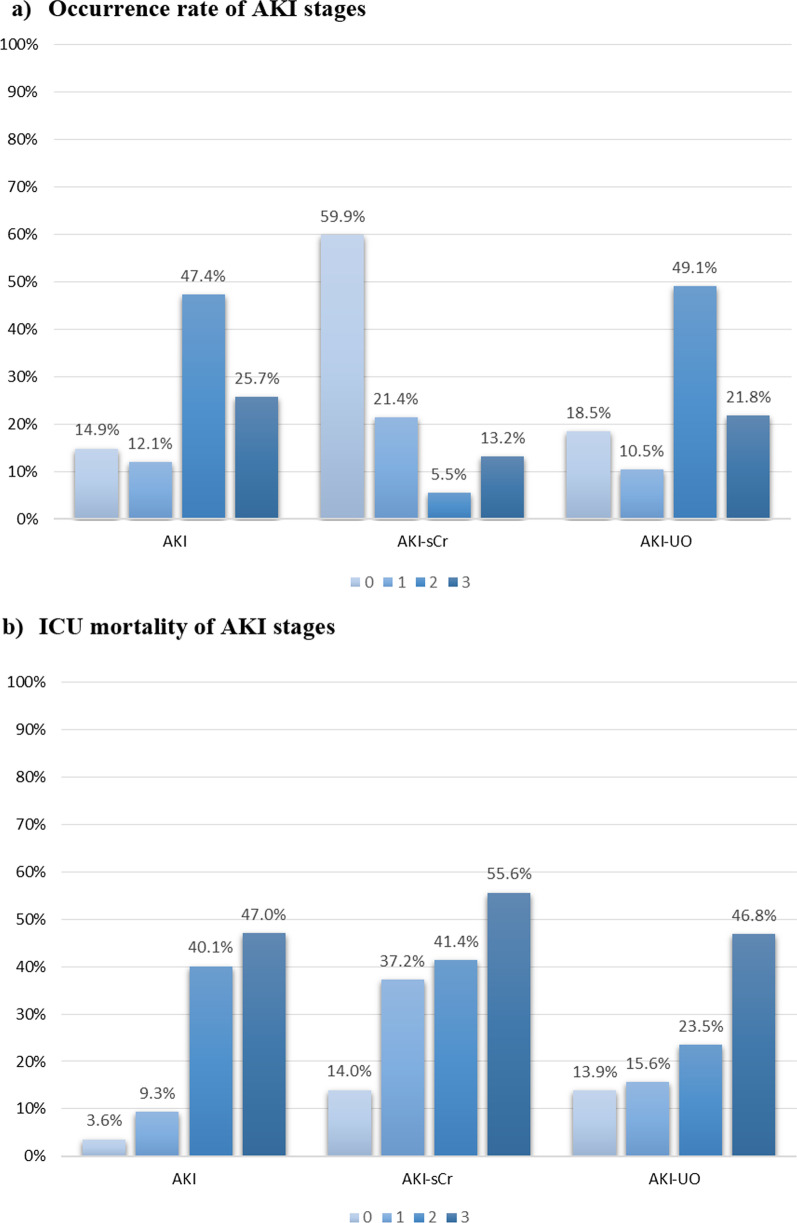
Occurrence rate and ICU mortality of AKI stages. **a** Occurrence rate and **b** ICU mortality of AKI stages defined according to the full KDIGO definition and its components AKI-sCr and AKI-UO. AKI = acute kidney injury, AKI-sCr = AKI based on creatinine criteria only, AKI-UO = AKI based on urine output criteria only. Statistical significance of comparison of ICU mortality in AKI-sCr versus AKI-UO stages: AKI stage 0: *p* = 0.002; AKI stage 1: *p* < 0.001; AKI stage 2: *p* = 0.001; AKI stage 3: *p* = 0.070

Of 1286 patients, 85.1% developed AKI (95% CI 83.2%, 87.1%). The maximum AKI stage was 1 in 12.1% of patients (95% CI 10.3%, 13.8%), stage 2 in 47.4% (95% CI 44.7%, 50.2%), stage 3 in 25.7% (95% CI 23.3%, 28.0%) (Fig. 2a); 9.8% of patients (95% CI 8.2%, 11.4%) were treated with KRT.

##### Risk factors for developing AKI

Patients with AKI were older, had a higher BMI and had more frequently diabetes and arterial hypertension compared to patients without AKI (Table 1). Baseline sCr was similar in both groups, while baseline estimated glomerular filtration rate (eGFR) was lower in the AKI group. AKI patients had higher severity-of-illness scores. Multivariable regression analysis showed an association between obesity and AKI (odds ratio (OR) 3.055, 95% CI 1.745, 5.347; *p* < 0.001) (Additional file 1: Table S5a, b, c). Other risk factors were age (OR 1.038, 95% CI 1.018, 1.059; *p* < 0.001) and invasive mechanical ventilation (IMV) on day of ICU admission (OR 2.182, 95% CI 1.144, 4.163; *p* = 0.018). Chronic kidney disease (CKD), hypertension, diabetes, APACHE II and vasoactive therapy at ICU admission were not associated with AKI.

#### Patient outcomes

##### Crude mortality

**Fig. 3:**
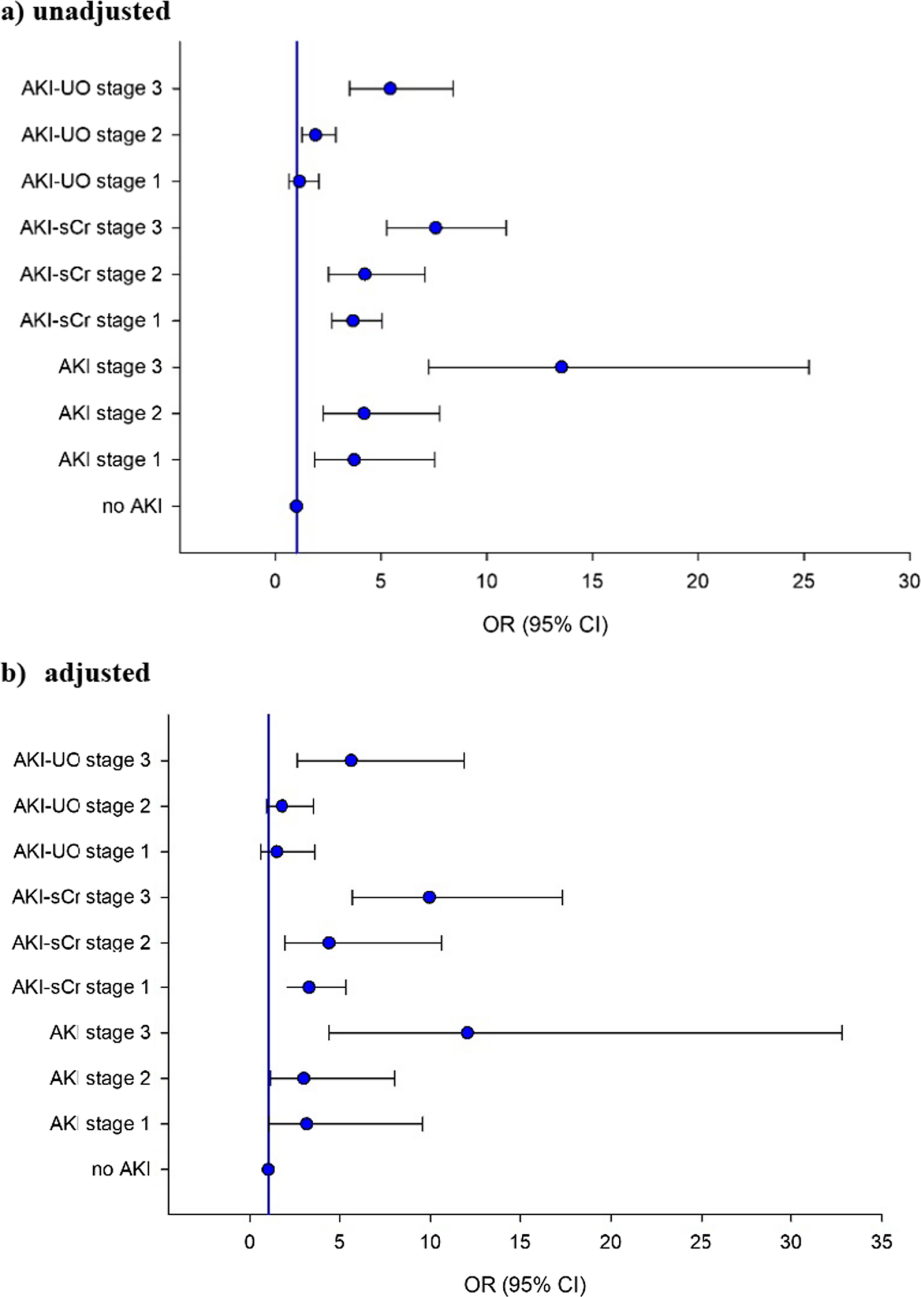
Association between ICU mortality and AKI stages. Association between ICU mortality and AKI stage according to serum creatinine and/or urine output: **a** unadjusted, **b** adjusted

Compared to patients without AKI, AKI patients had higher ICU, hospital and 30-day mortality (Fig. 2b; Table 2). Increasing AKI stages had increasing ORs for ICU mortality (stage 1: OR 3.729, 95% CI 1.843, 7.544; stage 2: OR 4.199, 95% CI 2.270, 7.768, stage 3: OR 13.537, 95% CI 7.260, 25.243) (Fig. 3a; Additional file 1: Table S6a, b). Patients on KRT had an increased ICU, hospital and 30-day mortality compared to patients not treated with KRT (*p* < 0.001) (Table 2).

**Table 2:** Patient and kidney outcomes according to different AKI categories and AKD

Mortality	ICU (*n* = 1286)	*P*	Hospital (*n* = 1282)	*P*	30-day (*n* = 807)	*P*
*(a) Patient outcomes*						
No AKI AKI	12 (6.3%) 322 (29.4%)	< 0.001	17 (8.9%) 350 (32.1%)	< 0.001	11 (18.3%) 297 (37.3%)	0.003
No AKI-sCr AKI-sCr	108 (14.0%) 226 (43.8%)	< 0.001	133 (17.3%) 234 (45.5%)	< 0.001	95 (27.0%) 195 (42.9%)	< 0.001
No AKI-UO AKI-UO	33 (13.9%) 300 (28.7%)	< 0.001	40 (16.9%) 326 (31.3%)	< 0.001	28 (27.5%) 261 (37.2%)	0.056
RR-AKI P-AKI	23 (18.4%) 311 (26.8%)	0.042	29 (23.2%) 338 (29.2%)	0.158	22 (36.1%) 268 (35.9%)	0.982
No AKD AKD	9 (5.7%) 325 (28.8%)	< 0.001	11 (6.9%) 356 (31.7%)	< 0.001	8 (17.8%) 282 (37.0%)	0.009
No KRT KRT	263 (22.7%) 71 (56.3%)	< 0.001	295 (25.5%) 72 (57.1%)	< 0.001	232 (33.9%) 58 (47.5%)	0.004
*Length of stay (days)*						
All No AKI AKI	11 (5.3, 22) 5 (2.8, 7.9) 12.1 (6.7, 24)	< 0.001	18 (7, 34) 13.5 (6, 22) 19 (7, 36)	< 0.001	/	/
*(b) Kidney outcomes*						
AKI		1095/1286 (85.1%)				
ICU day start AKI (d)		2 (1, 2)				
ICU day AKI max (d)		2 (2, 4)				
Duration of AKI (d)		5 (3, 9)				
RR-AKI		125 (11.4%)				
AKD (*n* = 1286) AKD–no AKI (*n* = 191) AKD–AKI (*n* = 1095)		1127 (87.6%) 32 (2.8% of all AKD) 1095 (97.2% of all AKD)				
KRT (*n* = 1286) CKRT IHD		126 (9.8%) 75 (59.5%) 51 (40.5%)				
KRT start (d of ICU/date)		7 (2, 12)				
KRT end (d of ICU/date)		17 (10, 22)				
Duration KRT (d) (*n* = 96)		8 (3, 14)				
Kidney recovery (ICU survivors) AKI (*n* = 773) AKI-sCr (*n* = 290) AKI-UO (*n* = 740) AKI-UO excl KRT (*n* = 725)		AKI-sCr vs AKI-UO: *p* < 0.001 369 (47.7%) 199 (68.6%) 407 (55.0%) 397 (54.8%)				
sCr@d21/ICU discharge (mg/dl) All ICU survivors (*n* = 952) No AKI (*n* = 179) AKI (*n* = 773)		no AKI versus AKI *p* = 0.266 0.73 (0.61, 0.93) 0.70 (0.65, 0.90) 0.72 (0.60, 0.95)				
KRT at 21 days or ICU discharge (*n* = 95)		64 (67.4%)				

*AKI* acute kidney injury, *AKI-sCr* AKI based on creatinine criteria only, *AKI-UO* AKI based on urine output criteria only, *RR-AKI* AKI with rapid reversal, *P-AKI* persistent AKI, *AKD* acute kidney disease, *KRT* kidney replacement therapy, *CKRT* continuous kidney replacement therapy, *IHD* intermittent hemodialysis, *ICU* intensive care unit, *excl* excluded, *sCr* serum creatinine, *d* day

Continuous variables are reported as median and interquartile ranges; categorical variables are reported as absolute numbers and frequencies

##### Adjusted risk for ICU mortality

After adjustment for covariates, all AKI stages were associated with ICU mortality (stage 1 OR: 3.179, 95% CI 1.054, 9.583; stage 2 OR: 3.024, 95% CI 1.139, 8.028; stage 3 OR: 12.039, 95% CI 4.418, 32.802) (Fig. 3b; Additional file 1: Table S6c, d). The model included age, baseline eGFR and comorbidities (arterial hypertension, COPD, diabetes, malignancy, immunodepression) as well as severity of illness at ICU admission (APACHE II score, PaO_2_/FiO_2_ and use of vasoactive therapy).

### Secondary outcomes

#### AKI occurrence and stages according to sCr or UO

AKI-sCr was present in 40.1% (95% CI 37.4%, 42.8%) and AKI-UO in 81.5% of all patients (95% CI 79.4%, 83.6%) (Table 2). Occurrence of AKI-sCr and AKI-UO stages is presented in Fig. 2a.

#### Patient outcomes

##### Crude mortality according to AKI-sCr or AKI-UO

In patients with AKI-sCr ICU, hospital and 30-day mortality were higher than in patients without AKI-sCr. This was similar for AKI-UO patients, except for 30-day mortality (Table 2). ICU mortality for AKI stages 1 and 2 was significantly higher when defined by AKI-sCr criteria compared to AKI-UO criteria (Fig. 2b; Table 3). All AKI-sCr stages and AKI-UO stage 2 and 3 were associated with ICU mortality in univariate analysis (Fig. 3a). Patients with AKI-sCr had higher ICU mortality (43.8% resp. 28.7%, *p* < 0.001) and hospital mortality (45.5% resp. 31.3%, *p* < 0.001) compared to patients with AKI-UO. A similar nonsignificant trend could be observed in 30-day mortality (42.9% resp. 37.2%, *p* = 0.054). Of patients not meeting AKI-sCr criteria, 75% had AKI-UO with a crude ICU mortality of 16.6% compared to 6.3% in the patients without any AKI (*p* < 0.001) (Table 3).

**Table 3:** AKI stages according to serum creatinine or urine output and ICU mortality

AKI stages	No AKI-UO	AKI-UO 1	AKI-UO 2	AKI-UO 3	Total
No AKI-sCr ICU mortality	190 12 **(6.3%)**	100 15 (15.0%)	409 68 (16.6%)	70 13 (18.6%)	769 **108 (14.0%)**
AKI-sCr 1 ICU mortality	29 10 (34.5%)	25 5 **(20.0%)**	150 52 (34.7%)	70 35 (50.0%)	274 **102 (37.2%)**^**1**^
AKI-sCr 2 ICU mortality	7 4 (57.1%)	6 0 (0%)	37 10 **(27.0%)**	20 15 (75.0%)	70 **29 (41.4%)**^**2**^
AKI-sCr 3 ICU mortality	11 7 (63.6%)	4 1 (25.0%)	34 18 (52.9%)	120 68 **(56.7%)**	169 **94 (55.6%)**^**3**^
Total ICU mortality	237 **33 (13.9%)**	135 **21 (15.6%)**^**1**^	630 **148 (23.5%)**^**2**^	280 **131 (46.8%)**^**3**^	1282 **(333) 26.0%**

Bold 1 (diagonal): ICU mortality in patients having AKI according to both criteria (sCr and UO). Bold 2 (row or column 'total'): ICU mortality per AKI stage according to the used criterium

Statistical significance of comparison of ICU mortality in AKI-sCr versus AKI-UO stages: ^1^AKI stage 1: *p* < 0.001; ^2^AKI stage 2: *p* = 0.001; ^3^AKI stage 3: *p* = 0.070

*AKI* acute kidney injury, *AKI-sCr* AKI based on creatinine criteria only, *AKI-UO* AKI based on urine output criteria only, *ICU* intensive care unit

##### Adjusted risk for ICU mortality according to AKI definition

After correction for covariates, all AKI-sCr stages and AKI-UO stage 3 were associated with ICU mortality (adjusted OR AKI-sCr stage 1: 3.319, 95% CI 5.026, 5.345; stage 2: 4,417, 95% CI 1.921, 10.621; stage 3: 9943, 95% CI 5.696, 17.355; adjusted OR AKI-UO stage 1: 1.474, 95% CI 0.597, 3.641; stage 2: 1.784, 95% CI 0.891, 3.570; stage 3: 5.636, 95% CI 2.676, 11.868) (Fig. 3b; Additional file 1: Table S6d). In a subanalysis of patients who developed AKI-UO but did not fulfill sCr criteria for AKI at any point, only AKI-UO stage 3 was associated with adjusted ICU mortality (Additional file 1: Table S6e).

##### Crude mortality according to AKD and rapid reversal of AKI

Patients who developed AKD had higher ICU, hospital and 30-day mortality compared to patients without AKD (Table 2). ICU mortality was higher in patients with persistent AKI than in patients with rapid reversal of AKI (Table 2).

##### Length of stay

Patients with AKI had a longer ICU and hospital LOS than patients without AKI (p < 0.001) (Table 2).

#### Kidney outcomes

AKI occurred early during ICU stay with a maximum AKI stage at 2 days after admission and a median duration of 5 days (IQR 3–9) (Table 2). In 11.5% of AKI patients (95% CI 9.6%, 13.4%), KRT was initiated starting at a median interval of 7 days (IQR 2–12.5) after admission with a median duration of 8 days (IQR 3–14). Continuous KRT (CKRT) was initiated in 59.5% and intermittent hemodialysis (IHD) in 40.5%.

We observed rapid reversal in 11.4% of AKI patients (95% CI 9.5%, 13.3%), whereas 88.6% had persistent AKI (95% CI 86.7%, 90.5%) and 87.6% developed AKD (95% CI 85.8%, 89.4%). Patients with rapid reversal had more AKI based on UO only, compared to patients with persistent AKI (Table 4). Kidney recovery at 21 days or ICU discharge, whichever came first, was observed in 47.4% of all survivors with AKI (95% CI 44.2%, 51.3%), in 68.6% when AKI-sCr was present (95% CI 63.3%, 74.0%), and in 55.0% in case of AKI-UO (95% CI 21.4%, 58.6%). Of all patients who received KRT, 67.4% were still on KRT at time of ICU discharge or at day 21 after ICU admission (95% CI 54.6%, 73.4%). There was no significant difference in kidney recovery between patients treated with CKRT compared to IHD. Crude patient and kidney outcomes according to KRT modality are outlined in Table 5. After correction for age, diabetes, arterial hypertension, APACHE II, PaO2/FiO2 and vasopressors at day 1, we observed a trend toward better survival in the patients who were treated with CKRT (aOR 0.211, 95% CI 0.044–1.007) compared to patients treated with IHD (Hosmer–Lemeshow 0.034).

**Table 4:** Rapid reversal of AKI according to fulfilled AKI criteria (sCr, UO or both)

AKI	sCr (*n* = 50)	UO (*n* = 579)	sCr + UO (*n* = 466)	*p* Value
P-AKI	32	484	454	< 0.001
(*n* = 970)	3.3%	50.1%	46.8%	
RR-AKI	18	95	12	
(*n* = 125)	14.4%	76.0%	9.6%	

*AKI* acute kidney injury, *P-AKI* persistent AKI, *RR-AKI* rapid reversal AKI, *UO* urine output, *sCr* serum creatinine, *KRT* kidney replacement therapy, *sCr* sCr criterion is fulfilled, *UO* UO criterion is fulfilled, *sCr* + *UO* both criteria are fulfilled to define AKI

**Table 5:** Crude patient and kidney outcomes according to KRT modality

	CKRT (*n* = 75)	IHD (*n* = 51)	*p* Value
ICU mortality	42/75 (56.0%)	29/51 (56.9%)	0.924
Hospital mortality	42/75 (56.0%)	30/51 (58.8%)	0.753
Kidney recovery (UO) in ICU survivors	20/33 (60.6%)	10/22 (45.5%)	0.269
Kidney recovery (sCr) in ICU survivors	14/33 (42.4%)	7/22 (31.8%)	0.428
Kidney recovery (KRT) in ICU survivors	22/33 (66.6%)	2/6 (33.3%)	0.123

*CKRT* continuous kidney replacement therapy, *IHD* intermittent hemodialysis, *ICU* intensive care unit, *UO* urine output, *sCr* serum creatinine, *KRT* kidney replacement therapy, *Kidney recovery (UO)* patient does not fulfill AKI-UO criteria, *Kidney recovery (sCr)* patient does not fulfill AKI-sCr criteria, *Kidney recovery (KRT)* patient is free of KRT

### Sensitivity analysis

As a sensitivity analysis, assessing risk factors for developing AKI and for ICU mortality was performed using only true baseline sCr which confirmed the results (Additional file 1: Table S5c, S6d).

### Second COVID-19 wave versus first wave

Occurrence of AKI was similar during the two waves, independent of the AKI criterion used (Additional file 1: Table S7). Rates of rapid reversal of AKI and AKD did not differ between the two waves. KRT was less frequently used during the second wave (11.9% vs. 8.3%, *p* = 0.035).

Patients during the second wave were significantly older (median 67 y vs. 70 y, *p* = 0.002) and had a lower baseline serum creatinine. They had a lower SOFA and APACHE II score at admission. During the second wave, corticosteroids were used during hospitalization in almost all patients, remdesivir was administered more frequently, and hydroxychloroquine was not used. At day 1 of ICU admission, high-flow oxygen was more frequently applied; mechanical ventilation was used less often as well as vasoactive drugs. ICU mortality was significantly higher in the second wave (21.2% vs. 29%; *p* = 0.002) in all patients and in patients with AKI (24.4% vs. 32.6%; *p* = 0.003) (Additional file 1: Table S8).

## Discussion

In this large multicenter cohort of 1286 patients with critical COVID-19, we found a high rate of AKI (85.1%) as defined by the 2012 KDIGO guidelines which occurred early in the disease course. KRT was used in 9.8% of patients. Older age, obesity, a higher APACHE II score and use of mechanical ventilation at day 1 of ICU stay were associated with an increased risk of AKI. After correction for confounders, all AKI stages were associated with ICU mortality.

In the majority of patients, AKI diagnosis was driven by UO criteria, while only 40% had AKI based upon a rise in sCr. The association between AKI and ICU mortality was more pronounced when AKI was diagnosed due to elevated sCr. In contrast, when staging was based upon UO, only AKI stage 3 was an independent predictor of ICU mortality.

Most patients developed persistent AKI and AKD. Kidney recovery was observed in almost half of surviving AKI patients. Rapid reversal of AKI yielded a better prognosis compared to patients with persistent AKI and AKD. Occurrence of AKI was similar during the first and the second COVID-19 wave in Belgium; ICU mortality was higher in the second COVID-19 wave in all patients and in AKI patients compared to the first wave.

Our study confirms that AKI is a frequent complication in critical COVID-19. The demonstrated AKI rate of 85% is markedly more than the 40–60% occurrence rate of AKI observed in a general ICU population [12–14]. We found that the median rate of AKI reported in critical COVID-19 was 55.8%, with a broad variation (Additional file 1: Table S1). Several studies in the USA also reported AKI in 60–80% of critical COVID-19 patients [6, 15–17]. This could even be an underestimation as some investigators defined AKI on sCr only [16, 17]. In a French cohort of 379 critical COVID-19 patients, half of patients had AKI in the first 7 days after ICU admission [18]. Lumlertgul et al. [3] observed AKI in 76% of 313 critical COVID-19 patients, which was more concordant with our findings. They used a time window of 14 days after ICU admission. In this cohort, we studied AKI occurrence based on the full KDIGO definition within the first 21 days of ICU admission which could be an explanation for the difference. A multicenter cohort from Wuhan found AKI-sCr in almost half of the 275 patients with critical COVID-19 when the entire hospital stay was evaluated [19]. This is in line with our findings based on sCr alone.

The pathophysiology of AKI in critical COVID-19 is multifactorial. Acute tubular injury is the most frequent histological finding; however, collapsing glomerulopathy and thrombotic microangiopathy have been observed in this population. Other rare findings such as anti-neutrophil cytoplasmic antibody vasculitis, anti-glomerular basement membrane disease and podocytopathies have been reported [7, 20, 21]. Endothelial damage as well as local and systemic inflammatory responses with complement activation might play a role. Renal tropism by SARS-CoV-2 with direct invasion of the kidney has been proposed but remains controversial [22]. In critical COVID-19, indirect factors such as the presence of hypoxemia, hypotension, hypo- or hypervolemia could also contribute to the development of AKI as well as the use of specific treatments such as mechanical ventilation with high positive end-expiratory pressure or high inspiration pressure and use of nephrotoxic drugs [7, 21].

Use of KRT in critical COVID-19 was low in our cohort, compared to some previous epidemiological studies which observed use of KRT in up to 44% of patients [6]. In our summary of data reported in the literature, the median number of patients treated with KRT was 24.5%, and 25% of studies reported that less than 16.3% of patients were treated with KRT (Additional file 1: Table S1). This may be explained by differences in patient characteristics and variation in practice patterns. The threshold to initiate KRT might vary among ICU physicians and centers. The decision to initiate KRT may have been more conservative compared to a general ICU population. In view of the infectious hazard, doctors seem to be more reluctant to expose themselves and other healthcare professionals to COVID-19 patients which might delay consultation and hence treatment as shown by Scherer et al. [23]. However, restrictive use of KRT has no impact on patient outcomes as shown in several large studies and a meta-analysis. [24–27]. We hypothesize that integration of STARRT-AKI conclusions in daily practice was the main driver for the high threshold to start KRT in this cohort [24].

In this study, all AKI stages were associated with increased ICU mortality, which is in line with some reports in critical COVID-19, but in contrast to most studies in general ICU patients [13, 15, 19, 28]. The relation between AKI and mortality was less pronounced when UO was used as the only diagnostic tool for AKI. Previously, the prognostic value of oliguria (UO < 0.5 ml/kg/h) has already been questioned in a general ICU population [29]. However, Kellum et al. showed that patients meeting both UO and sCr criteria for AKI in critically ill had worse outcomes compared to patients with AKI diagnosis based on one criterion. They advocate the absolute need for UO in diagnosing AKI [30]. This is supported by the observation of Macedo et al. that UO was a sensitive and early marker for AKI and associated with adverse outcomes in critically ill patients [31].

In our cohort, we observed that 75% of patients not meeting AKI-sCr criteria had AKI-UO, with a higher crude ICU mortality compared to patients without any AKI. In a subanalysis of patients with AKI-UO not fulfilling sCr criteria, only AKI-UO stage 3 was associated with ICU mortality after adjustment for covariates. This suggests that severe AKI-UO identified a relevant subset of AKI patients, similar to findings in the general ICU population [30].

The finding that oliguric AKI does not necessarily lead to worse prognosis in critical COVID-19 may be explained by the hypothesis that a decrease in UO rather reflects a functional change in kidney function without structural damage, whereas changes in sCr could be more compatible with structural changes. Biomarkers could play a role to differentiate the several phenotypes of AKI [32]. A decreased UO might be an early indicator of hypovolemia, e.g., secondary to insensible losses caused by fever and elevated work of breathing, which could precede biochemical changes and kidney damage when these adaptations to altered hemodynamics are persisting.

The high rate of oliguria in this cohort might be related to the conservative fluid management which was initially advised in treatment of critical COVID-19 [1]. This hypothesis could not be evaluated in this cohort. On the one hand, we assumed that not only fluid dynamics during ICU stay, but also fluid management before ICU admission might have influenced AKI occurrence since AKI occurred early during ICU stay and patients were already admitted to the hospital for a median duration of 8 days before entering the ICU. Fluid balances before ICU admission could not be obtained. On the other hand, fluid balance might not reflect the effective circulating volume. Standardized assessment of the intravascular volume status was not possible in our cohort since invasive hemodynamic monitoring was seldom used.

Rapid reversal of AKI yielded a better prognosis. Patients with rapid reversal had more AKI based on UO only, compared to patients with persistent AKI, which supports the hypothesis that compared to persistent AKI, a greater proportion of rapid reversal AKI could be caused by functional changes, i.e., hypovolemia or low effective circulating volume, leading to oliguria. Optimizing hemodynamics is key in treating these patients.

The high proportion of obese patients in the AKI group (one-third) and the use of actual body weight for calculation of UO as mL/kg/h could partially explain the high occurrence of oliguric AKI. It has been suggested to use adjusted body weight to classify oliguric AKI to avoid over-classification of AKI in obese patients [33]. This concept needs further validation.

Patients with critical COVID-19 and AKD had worse outcomes than patients without AKD, which is similar to findings in the general ICU population [34]. The occurrence of AKD was very high during ICU stay. According to the 2021 KDIGO definition, the diagnosis of AKD is met when AKI criteria are fulfilled, irrespective of the 7-d time window [10]. Thus, AKI is considered a subgroup of AKD which explains this high rate.

Almost 50% of surviving patients demonstrated kidney recovery at 21 days or at ICU discharge. Kidney recovery rate is probably higher at longer follow-up as has been demonstrated by Lumlertgul and colleagues who observed kidney recovery in 90.9% of survivors at 90 days [3]. These findings confirm that AKD is often a reversible condition which compasses patient groups with different disease trajectories [10].

When comparing the first and the second COVID-19 wave in Belgium, occurrence of AKI was similar. The crude ICU mortality as well as AKI-associated mortality was higher in the second versus the first wave. First, differences between measured confounders could certainly play a role. In the second wave, patients admitted to ICU were older compared to the first wave. More use of HFO during the second wave with a potential intubation delay might also have contributed to this difference. However, the hypothesis that HFO delays intubation in critical COVID-19 is not supported by a recent meta-analysis [35]. Second, mortality was most likely influenced by unmeasured confounders, such as the strain on the healthcare system, which was more pronounced in the second wave in Belgium with less postponement of regular care which could have led to a compromised COVID capacity and expertise, in comparison with the first wave (Additional file 1: Figure S1). Furthermore, studies have demonstrated that bacterial surinfections and fungal infections are frequent in patients with critical COVID-19 [36–38]. We did not compare the occurrence of ICU-acquired infections in the first versus the second wave which could also have contributed to ICU mortality.

## Strengths

We conducted a multicenter study including seven large ICUs in Flanders. Using electronically recorded data, we collected a large dataset including hourly UO during 21 days of ICU admission which made it possible to detect AKI patterns and differences in diagnosis depending on the used AKI criterion in critical COVID-19. To our knowledge, this is one of the largest multicenter cohort studies on COVID-19-associated AKI in critically ill patients using the full KDIGO definition of AKI and the first study exploring the differences between AKI defined by UO or by sCr as well as the relation to prognosis in COVID-19-associated AKI. In addition, we also explored the occurrence and outcomes of rapid reversal of AKI, persistent AKI and AKD.

## Limitations

This study was retrospective in design; hence, we could only explore association and not causality. Estimation of the volume status of our patients was not possible in this retrospective cohort. Furthermore, patients were admitted to the hospital for over 1 week before ICU admission and developed AKI early in their ICU course. We therefore did not report fluid balances; hence, the association between fluid balance and AKI could not be explored.

Follow-up at 30 days was only available in two-thirds of patients, and long-term follow-up data at, for example, 90 days was unavailable which limits conclusions about long-term outcomes. Kidney outcomes were pragmatically assessed until up to 21 days after ICU admission; therefore, the presented rates of AKD and kidney recovery as well as duration of KRT could be underestimated.

Differences in treatment of COVID-19 patients within centers could be present which is inherent to the multicentric design. We included academic and non-academic centers as well as several types of ICU (medical, surgical, mixed). Since we aimed to give a global picture of COVID-associated AKI in Belgium, and given the fact that some centers had more than 1 ICU, we did not correct for a potential center effect in our analysis. Related to the level of care in most participating hospitals, potential referral bias could have resulted in a higher number of patients with more severe illness being admitted to these centers compared to non-referral centers which could lead to a higher rate of AKI.

## Conclusions

In this large cohort of critical COVID-19, AKI occurred in 85% of patients. This was largely driven by the high occurrence rate of AKI defined by UO criteria. KRT was used in 1 in 10 patients. All AKI stages were associated with ICU mortality. AKI defined by UO criteria had better patient outcomes compared to AKI defined by sCr criteria. Kidney recovery at ICU discharge or at 21 days of ICU admission was present in almost half of surviving AKI patients.

## Supplementary Information

**Additional file 1.** COVID-AKI-Belgium.

## Data Availability

All data and materials generated during the current study are available from the corresponding author on reasonable request.

## Abbreviations

ACEI: Angiotensin-converting enzyme inhibitors
AKD: Acute kidney disease
AKI: Acute kidney injury
AKI-sCr: AKI based on creatinine criteria only
AKI-UO: AKI based on urine output criteria only
ARBs: Angiotensin receptor blockers
AUC-ROC: Area under the curve of the receiver operating characteristic curve
BMI: Body mass index
CI: Confidence interval
CKD: Chronic kidney disease
CKRT: Continuous kidney replacement therapy
COPD: Chronic obstructive pulmonary disease
COVID: Coronavirus disease
CRP: C-reactive protein
CV: Cardiovascular
d: Day
ECMO: Extracorporeal membrane oxygenation
eGFR: Estimated glomerular filtration rate
FiO_2_: Fraction of inspired oxygen
GM-CSF: Granulocyte-macrophage colony-stimulating factor
HFO: High-flow oxygen
hosp: Hospital
ICU: Intensive care unit
IHD: Intermittent hemodialysis
IL: Interleukin
Immuno: Immune compromised
IMV: Invasive mechanical ventilation
KDIGO: Kidney disease: improving global outcomes
KRT: Kidney replacement therapy
LOS: Length of stay
MAP: Mean arterial pressure
MDRD: Modification of diet in renal disease
mmHg: Millimeters of mercury
NIV: Noninvasive ventilation
NSAIDs: Nonsteroidal anti-inflammatory drugs
PaO2: Arterial oxygen pressure
y: Year
