# Supplementary material for: Acute kidney injury in critical COVID-19: a multicenter cohort analysis in seven large hospitals in Belgium
Source: Crit Care. 2022 Jul 25;26:225. doi: 10.1186/s13054-022-04086-x (PMC9310674; doi:10.1186/s13054-022-04086-x)
Supplement: Supplementary file 1 — Additional file 1. COVID-AKI-Belgium. [file 13054_2022_4086_MOESM1_ESM.docx]

**Acute Kidney Injury in critical COVID-19: a multicentre cohort analysis in 7 large hospitals in Belgium.**

**Additional file**

Table of contents

[Introduction: Additional table 1: overview of studies reporting on AKI in critical COVID-19 patients 4](#_Toc105590655)

[Methods: Additional table 2: STROBE Statement—Checklist of items that should be included in reports of cohort studies 11](#_Toc105590656)

[Methods: Definitions used 13](#_Toc105590657)

[Methods: Additional table 3A: Definitions of Acute Kidney Injury (AKI), Acute Kidney Disease (AKD), Chronic Kidney Disease (CKD) (1, 6) 14](#_Toc105590658)

[Methods: Additional table 3B: AKI stages 15](#_Toc105590659)

[Methods: Additional figure 1: Number of beds occupied by patients with COVID-19 in ICUs in Belgium 15](#_Toc105590660)

[Results: Additional table 4: eGFR based on true baseline creatinine, MDRD back-calculation or lowest creatinine during hospital stay 16](#_Toc105590661)

[Results: Additional table 5a: Risk factors for developing AKI (multivariable logistic regression analysis) 17](#_Toc105590662)

[Results: Additional table 5b: Risk factors for developing AKI based on creatinine criteria only (multivariable logistic regression analysis) 17](#_Toc105590663)

[Results: Additional table 5c: Risk factors for developing AKI in the cohort of patients with known baseline serum creatinine (multivariable logistic regression analysis) 18](#_Toc105590664)

[Results: Additional table 6a: Variables associated with ICU mortality 19](#_Toc105590665)

[Results: Additional table 6b: Variables associated with ICU mortality (logistic regression analysis) – unadjusted 23](#_Toc105590666)

[Results: Additional table 6c: Variables associated with ICU mortality (multivariable logistic regression analysis) – adjusted odds ratios 23](#_Toc105590667)

[Results: Additional table 6d: Variables associated with ICU mortality (multivariable logistic regression analysis) –odds ratios for adjusted models in cohorts defined by the KDIGO AKI definition, the variants based on AKI-sCR or AKI-UO, and in the cohort with known baseline serum creatinine 24](#_Toc105590668)

[Results: Additional table 6e: Variables associated with ICU mortality (multivariable logistic regression analysis) in patients with AKI-UO who do not fulfill AKI-sCr criteria – adjusted odds ratios 26](#_Toc105590669)

[Results: Additional table 7: Comparison of patient characteristics in the first versus the second COVID-19 wave 27](#_Toc105590670)

[Results: Additional table 8: ICU mortality according to AKI Stage: comparison between first and second COVID-19 wave 29](#_Toc105590671)

[Acknowledgements 30](#_Toc105590672)

[References 33](#_Toc105590673)

# **Introduction**: Additional table 1: overview of studies reporting on AKI in critical COVID-19 patients

| **Study** | **Design** | **Number of centres, country** | **Inclusion criteria** | **Number of patients** | **AKI definition** | **AKI** | **KRT** | **Mortality**  **AKI** | **Mortality KRT** |
| --- | --- | --- | --- | --- | --- | --- | --- | --- | --- |
| Abdallah et al[10] | Prospective cohort | 1, Kuwait | ICU | 198 | KDIGO | 30.8% | 24.2% | 39% | - |
| Alser et al.[11] | Prospective cohort | 1, USA | ICU | 235 | KDIGO, creatinine criteria | 69.8% | 23.8% | - | - |
| Bayrakci et al[9] | Retrospective cohort | Multicentre, Turkey | ICU | 328 | KDIGO, creatinine criteria | 26.8% | 4.9% | 48.9%. | - |
| Chaibi et al.[5] | Retrospective cohort | 9, France/Spain | ICU & ARDS | 211 | KDIGO | 58% | 14% | - | - |
| Chan et al.[8] | Retrospective  cohort | 5, NY (USA) | ICU | 976 | KDGIO, creatinine criteria | 76% | 32% | 42% | - |
| Chand et al.[12] | Retrospective  cohort | 1, NY, USA | ICU | 300 | KDIGO | 76.7% | 44.3% | 60% | 61.7% |
| Cheng et al.[13] | Retrospective Cohort | 1, China | ICU | 119 | KDIGO | 42.8% |  | 79.4% | - |
| Doher et al.[6] | Retrospective cohort | 6, Brazil | ICU | 207 | KDIGO | 50.2% | 17% | 23.8% | - |
| Geri et al.[14] | Retrospective cohort | 4, France | ICU | 379 | KDIGO, creatinine, urinary output in the first 24h | 52% | 19.5% | 37.4% | - |
| Ghosn et al.[15] | Retrospective cohort | 1, UAE | ICU | 110 | KDIGO stages 2 & 3 | AKI 2-3: 45.4% | 24.5% | AKI 2-3: 52% | - |
| Grimaldi et al.[16] | Retrospective cohort | 21, Belgium, France | ICU | 414 |  | 55.8% | 20.2% | 55% | 79% |
| Hittesdorf et al.[17] | Retrospective cohort | 1,USA | Provisional ICU in the OR | 116 | KDIGO | 65.5% | 38.8% | 46.1% | 40.0% |
| Costa et al.[18] | Retrospective cohort | 1, Brazil | ICU | 102 | KDIGO | 55.9% |  | 33.3% |  |
| Lumlertgul et al.[3] | Retrospective cohort | 1, UK | ICU | 313 | KDIGO, creatinine, urinary output (adjusted body weight) | 76.7% | 31.9% | 34%  al recovery at discharge 81.6%, at 90d 90.9%; MAKE90 42.4% |  |
| Elkholi et al.[19] | Retrospective cohort | 1, Dubai | ICU & Mechanical Ventilation | 198 | KDIGO | 65.1% | 17.2% | 65.9% | 61.5% |
| Naar et al.[20] | Prospective cohort | 1, USA | ICU | 206 | KDIGO | 71.8% | 22.3% | - | - |
| Pineiro et al.[21] | Prospective cohort | 1, Spain | ICU | 237 | AKIN stages 2 & 3 | AKI 2-3: 21.9% | 6.3% | 51.9% | - |
| Sang et al.[7] | Retrospective cohort | 2, China | ICU | 210 | KDIGO | 43.8% | 24.8% | 44.3% | - |
| Wang et al.[2] | Retrospective cohort | 13, China | ICU & ARDS | 275 | KDIGO, creatinine criteria | 49.5% | 13.5% | 80.1% | - |
| Xu et al.[4] | Retrospective  cohort | 19, China | ICU | 671 | KDIGO | 39.2% | 33% | 72% | - |
| Yakar et al.[22] | Retrospective cohort | 1, Turkey | ICU | 249 | KDIGO | 55.8% | 26.1% | 89.9% | 89.2% |
| Yu et al.[23] | Prospective cohort | China | ICU | 226 | KDIGO | 25.2% | 10.6% | - | - |
| **Min, Max** |  |  |  | **102 , 976** |  | **25.2%, 76.7%** | **4.9%, 44.3%** | **23.8%, 89.9%** | **40.0%, 89.2%** |
| **Median**  **(25%, 75%)** |  |  |  | **237**  **(198, 313)** |  | **55.8%**  **(43.6%, 66.6%)** | **24.5%**  **(16.3%, 32.3)** | **49.0%**  **(38.6%, 67.4%)** | **61.7%**  **(61.5%, 79.0%)** |

We conducted a search in PubMed with the following search term “COVID-19 AND (ICU OR Intensive care OR critical care) AND (AKI OR Acute kidney Injury) AND (Incidence OR occurrence)”. We included studies on ICU patients who had confirmed COVID-19 or more, and reported on AKI stages, and defined AKI according to the KDIGO or AKIN definition, and who reported on 100 adult patients or more. We excluded studies which only included patients on KRT.

**References**

1. Geri G, Ferrer L, Tran N, et al (2021) Cardio-pulmonary-renal interactions in ICU patients. Role of mechanical ventilation, venous congestion and perfusion deficit on worsening of renal function: Insights from the MIMIC-III database. J Crit Care. https://doi.org/10.1016/j.jcrc.2021.03.013

2. Wang F, Ran L, Qian C, et al (2020) Epidemiology and Outcomes of Acute Kidney Injury in COVID-19 Patients with Acute Respiratory Distress Syndrome: A Multicenter Retrospective Study. Blood Purificat 1–7. https://doi.org/10.1159/000512371

3. Lumlertgul N, Pirondini L, Cooney E, et al (2021) Acute kidney injury prevalence, progression and long-term outcomes in critically ill patients with COVID-19: a cohort study. Ann Intensive Care 11:123. https://doi.org/10.1186/s13613-021-00914-5

4. Xu J, Xie J, Du B, et al (2020) Clinical Characteristics and Outcomes of Patients With Severe COVID-19 Induced Acute Kidney Injury. J Intensive Care Med 36:319–326. https://doi.org/10.1177/0885066620970858

5. Chaibi K, Dao M, Pham T, et al (2020) Severe Acute Kidney Injury in Patients with COVID-19 and Acute Respiratory Distress Syndrome. Am J Resp Crit Care 202:1299–1301. https://doi.org/10.1164/rccm.202005-1524le

6. Doher MP, Carvalho FRT de, Scherer PF, et al (2021) Acute Kidney Injury and Renal Replacement Therapy in Critically Ill COVID-19 Patients: Risk Factors and Outcomes: A Single-Center Experience in Brazil. Blood Purificat 50:520–530. https://doi.org/10.1159/000513425

7. Sang L, Chen S, Zheng X, et al (2020) The incidence, risk factors and prognosis of acute kidney injury in severe and critically ill patients with COVID-19 in mainland China: a retrospective study. Bmc Pulm Med 20:290. https://doi.org/10.1186/s12890-020-01305-5

8. Chan L, Chaudhary K, Saha A, et al (2020) AKI in Hospitalized Patients with COVID-19. J Am Soc Nephrol 32:ASN.2020050615. https://doi.org/10.1681/asn.2020050615

9. Bayrakci N, Özkan G, Şakaci M, et al (2022) The incidence of acute kidney injury and its association with mortality in patients diagnosed with COVID‐19 followed‐up in intensive care unit. Ther Apher Dial. https://doi.org/10.1111/1744-9987.13790

10. Abdallah E, Helal BA, Asad R, et al (2021) Incidence and Outcomes of Acute Kidney Injury in Critically Ill Patients with Coronavirus Disease 2019. Saudi J Kidney Dis Transplant 32:84. https://doi.org/10.4103/1319-2442.318551

11. Alser O, Mokhtari A, Naar L, et al (2021) Multisystem outcomes and predictors of mortality in critically ill patients with COVID-19: Demographics and disease acuity matter more than comorbidities or treatment modalities. J Trauma Acute Care 90:880–890. https://doi.org/10.1097/ta.0000000000003085

12. Chand S, Kapoor S, Orsi D, et al (2020) COVID-19-Associated Critical Illness-Report of the First 300 Patients Admitted to Intensive Care Units at a New York City Medical Center. J Intensive Care Med 35:963–970. https://doi.org/10.1177/0885066620946692

13. Cheng Y, Zhang N, Luo R, et al (2021) Risk Factors and Outcomes of Acute Kidney Injury in Critically Ill Patients with Coronavirus Disease 2019. Kidney Dis 7:111–119. https://doi.org/10.1159/000512270

14. Geri G, Darmon M, Zafrani L, et al (2021) Acute kidney injury in SARS-CoV2-related pneumonia ICU patients: a retrospective multicenter study. Ann Intensive Care 11:86. https://doi.org/10.1186/s13613-021-00875-9

15. Ghosn M, Attallah N, Badr M, et al (2021) Severe Acute Kidney Injury in Critically Ill Patients with COVID-19 Admitted to ICU: Incidence, Risk Factors, and Outcomes. J Clin Medicine 10:1217. https://doi.org/10.3390/jcm10061217

16. Grimaldi D, Aissaoui N, Blonz G, et al (2020) Characteristics and outcomes of acute respiratory distress syndrome related to COVID-19 in Belgian and French intensive care units according to antiviral strategies: the COVADIS multicentre observational study. Ann Intensive Care 10:131. https://doi.org/10.1186/s13613-020-00751-y

17. Hittesdorf E, Panzer O, Wang D, et al (2021) Mortality and renal outcomes of patients with severe COVID-19 treated in a provisional intensive care unit. J Crit Care 62:172–175. https://doi.org/10.1016/j.jcrc.2020.12.012

18. Costa RL da, Sória TC, Salles EF, et al (2021) Acute kidney injury in patients with Covid-19 in a Brazilian ICU: incidence, predictors and in-hospital mortality. Jornal Brasileiro De Nefrologia 43:349–358. https://doi.org/10.1590/2175-8239-jbn-2020-0144

19. Elkholi MH, Alrais ZF, Algouhary AR, et al (2021) Acute kidney injury in ventilated patients with coronavirus disease-2019 pneumonia: A single-center retrospective study. Int J Critical Illn Inj Sci 11:123–133. https://doi.org/10.4103/ijciis.ijciis_194_20

20. Naar L, Langeveld K, Moheb ME, et al (2020) Acute Kidney Injury in Critically-ill Patients With COVID-19: A Single-center Experience of 206 Consecutive Patients. Ann Surg Publish Ahead of Print:e280–e281. https://doi.org/10.1097/sla.0000000000004319

21. Piñeiro GJ, Molina-Andújar A, Hermida E, et al (2020) Severe acute kidney injury in critically ill COVID-19 patients. Journal of Nephrology 1–9. https://doi.org/10.1007/s40620-020-00918-7

22. YAKAR MN, ERGAN B, ERGÜN B, et al (2021) Clinical characteristics and risk factors for 28-day mortality in critically ill patients with COVID-19: a retrospective cohort study. Turk J Med Sci 51:2285–2295. https://doi.org/10.3906/sag-2104-356

23. Yu Y, Xu D, Fu S, et al (2020) Patients with COVID-19 in 19 ICUs in Wuhan, China: a cross-sectional study. Crit Care 24:219. https://doi.org/10.1186/s13054-020-02939-x

Methods: Additional table 2: STROBE Statement—Checklist of items that should be included in reports of cohort studies

|  | Item No | Recommendation | Page No |
| --- | --- | --- | --- |
| **Title and abstract** | 1 | (*a*) Indicate the study’s design with a commonly used term in the title or the abstract | 1 |
|  |  | (*b*) Provide in the abstract an informative and balanced summary of what was done and what was found |  |
| Introduction | | | |
| Background/rationale | 2 | Explain the scientific background and rationale for the investigation being reported | 2 |
| Objectives | 3 | State specific objectives, including any prespecified hypotheses | 2 |
| Methods | | | |
| Study design | 4 | Present key elements of study design early in the paper | 3 |
| Setting | 5 | Describe the setting, locations, and relevant dates, including periods of recruitment, exposure, follow-up, and data collection | 3 |
| Participants | 6 | (*a*) Give the eligibility criteria, and the sources and methods of selection of participants. Describe methods of follow-up | 3 |
|  |  | (*b*) For matched studies, give matching criteria and number of exposed and unexposed |  |
| Variables | 7 | Clearly define all outcomes, exposures, predictors, potential confounders, and effect modifiers. Give diagnostic criteria, if applicable | 3-4 |
| Data sources/ measurement | 8* | For each variable of interest, give sources of data and details of methods of assessment (measurement). Describe comparability of assessment methods if there is more than one group | 4 |
| Bias | 9 | Describe any efforts to address potential sources of bias | 4 |
| Study size | 10 | Explain how the study size was arrived at | 3 |
| Quantitative variables | 11 | Explain how quantitative variables were handled in the analyses. If applicable, describe which groupings were chosen and why | 4 |
| Statistical methods | 12 | (*a*) Describe all statistical methods, including those used to control for confounding | 4 |
|  |  | (*b*) Describe any methods used to examine subgroups and interactions |  |
|  |  | (*c*) Explain how missing data were addressed |  |
|  |  | (*d*) If applicable, explain how loss to follow-up was addressed |  |
|  |  | (*e*) Describe any sensitivity analyses |  |
| Results | | |  |
| Participants | 13* | (a) Report numbers of individuals at each stage of study—eg numbers potentially eligible, examined for eligibility, confirmed eligible, included in the study, completing follow-up, and analysed | Fig 1;  P 6 |
|  |  | (b) Give reasons for non-participation at each stage |  |
|  |  | (c) Consider use of a flow diagram | Fig 1 |
| Descriptive data | 14* | (a) Give characteristics of study participants (eg demographic, clinical, social) and information on exposures and potential confounders | P6 -9; table 1 |
|  |  | (b) Indicate number of participants with missing data for each variable of interest |  |
|  |  | (c) Summarise follow-up time (eg, average and total amount) |  |
| Outcome data | 15* | Report numbers of outcome events or summary measures over time | P6-9 |

| Main results | 16 | (*a*) Give unadjusted estimates and, if applicable, confounder-adjusted estimates and their precision (eg, 95% confidence interval). Make clear which confounders were adjusted for and why they were included | P6-9;  Table 2,3 Fig 3 |
| --- | --- | --- | --- |
|  |  | (*b*) Report category boundaries when continuous variables were categorized |  |
|  |  | (*c*) If relevant, consider translating estimates of relative risk into absolute risk for a meaningful time period |  |
| Other analyses | 17 | Report other analyses done—eg analyses of subgroups and interactions, and sensitivity analyses | P6-9 additional tables |
| Discussion | | | |
| Key results | 18 | Summarise key results with reference to study objectives | P10 |
| Limitations | 19 | Discuss limitations of the study, taking into account sources of potential bias or imprecision. Discuss both direction and magnitude of any potential bias | P13 |
| Interpretation | 20 | Give a cautious overall interpretation of results considering objectives, limitations, multiplicity of analyses, results from similar studies, and other relevant evidence | P10-13 |
| Generalisability | 21 | Discuss the generalisability (external validity) of the study results | P10-13 |
| Other information | | | |
| Funding | 22 | Give the source of funding and the role of the funders for the present study and, if applicable, for the original study on which the present article is based | P 14 |

*Give information separately for exposed and unexposed groups.

**Note:** An Explanation and Elaboration article discusses each checklist item and gives methodological background and published examples of transparent reporting. The STROBE checklist is best used in conjunction with this article (freely available on the Web sites of PLoS Medicine at http://www.plosmedicine.org/, Annals of Internal Medicine at http://www.annals.org/, and Epidemiology at http://www.epidem.com/). Information on the STROBE Initiative is available at http://www.strobe-statement.org.

# **Methods**: Definitions used

Acute Kidney Injury (AKI) was defined according to the 2012 KDIGO criteria and further classified as rapid reversal (duration <48-h) or persistent AKI (≥48-h) according to the ADQI 16 work group consensus.(1-3) (Additional table 1) All patients had a bladder catheter. Urine output was mostly registered every hour. An algorithm was used to diagnose AKI based on at least 6 consecutive hours of oliguria.

Baseline serum creatinine (sCr) was determined as serum creatinine concentration available in the period 7-d to 1-y before ICU admission and after clinical adjudication. When this was not available or did not reflect true baseline kidney function (e.g. patients who already had AKI), we estimated baseline creatinine as the lowest value of a) the derived MDRD value as suggested by ADQI and KDIGO in patients without a diagnosis of chronic kidney disease (CKD) (4) or b) the lowest creatinine measured during hospital admission after exclusion of serum sCr values obtained during KRT. (4, 5)

Acute Kidney Disease (AKD) was defined according to the 2021 KDIGO recommendation. (Additional table 1) (6) AKI and AKD were pragmatically assessed up to day 21 of ICU admission. Kidney recovery was determined at 21 days or at ICU discharge if this occurred before 21 days, and defined as not meeting the KDIGO criteria (UO and/or sCr) for AKI or in case of KRT, free of KRT. (2, 7)

Severity of illness was assessed at ICU admission and at time of AKI diagnosis using the APACHE II, SAPS II, and SOFA scores.(8-10)

Immunocompromised status was defined according to the European Organization for Research and Treatment of Cancer and the Mycoses Study Group Education and Research Consortium. (11)

Obesity was defined as body mass index (BMI) of 30 kg/m^2^ and greater.(12)

According to the epidemiological situation in Belgium, the first COVID-19 wave was defined as ICU admission from February 1st until August 31st 2020 and the second wave September 1st 2020 until January 31st 2021 (Additional figure 1).(13)

Methods: Additional table 3A: Definitions of Acute Kidney Injury (AKI), Acute Kidney Disease (AKD), Chronic Kidney Disease (CKD) (1, 6)

| ***Definitions*** | **AKI** | **AKD** | **CKD** |
| --- | --- | --- | --- |
| **Duration** | Within 7 days | ≤ 3 months | >3 months |
| **Functional criteria** | sCr ↑0.3 mg/dl over 48 hours or > 50% within 7 days or UO of <0.5ml/kg/h for > 6 hours | AKI or GFR < 60 ml/min/1.73m² or decrease in GFR by ≥ 35% or increase in sCr by > 50% | GFR < 60 ml/min/1.73m² |
| **Structural criteria** | Not defined | Marker of kidney damage | Marker of kidney damage |

#

# Methods: Additional table 3B: AKI stages

| **AKI Classification** | | |
| --- | --- | --- |
| **AKI stage** | **Serum creatinine** | **Urinary output** |
| **1** | ≥ 1.5-1.9 x baseline or > 0.3 mg/dl | <0.5 ml/kg/hour for 6-12 hours |
| **2** | ≥ 2.0 – 2.9 x baseline | <0.5 ml/kg/hour for ≥ 12 hours |
| **3** | ≥ 3 x baseline or ≥ 4 mg/dl  or KRT | <0.3 mg/kg/hours for ≥ 24 hours or anuria for ≥ 12 hours |

Legend: AKI = acute kidney injury, KRT = kidney replacement therapy

Methods: Additional figure 1: Number of beds occupied by patients with COVID-19 in ICUs in Belgium

Figure from Sciensano, the Belgian Research Institute for public and animal health (20)


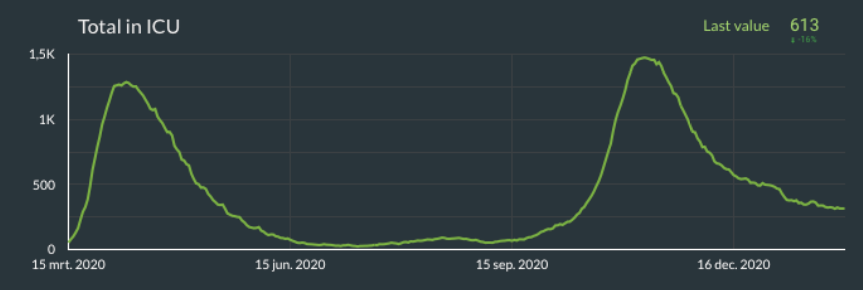


# Results: Additional table 4: eGFR based on true baseline creatinine, MDRD back-calculation or lowest creatinine during hospital stay

|  | **All (n = 1286)** | **No AKI**  **(n = 191)** | **AKI**  **(n = 1095)** | **P-value** |
| --- | --- | --- | --- | --- |
| **True baseline sCr** | 889 (69.1%) | 136 (71.2%) | 753 (68.8%) | 0.709 |
| **MDRD back-calculation** | 28 (2.2%) | 3 (1.6%) | 25 (2.3%) |  |
| **Lowest sCr Hosp** | 369(28.7%) | 52 (27.2%) | 317 (28.9%) |  |

Legend: AKI = acute kidney injury; sCr = serum creatinine, MDRD = Modification of Diet in Renal Disease, Hosp = hospital stay

# Results: Additional table 5a: Risk factors for developing AKI (multivariable logistic regression analysis)

| **Variable** | **OR** | **95% CI** | **P** |
| --- | --- | --- | --- |
| **Age** | 1.038 | 1.018, 1.059 | **<0.001** |
| **BMI>30** | 3.055 | 1.745, 5.347 | **<0.001** |
| **CKD** | 1.252 | 0.613, 2.557 | 0.537 |
| **Hypertension** | 0.777 | 0.465, 1.299 | 0.335 |
| **Diabetes** | 1.227 | 0.690, 2.179 | 0.486 |
| **APACHE II** | 1.038 | 0.995, 1.083 | **0.083** |
| **IMV @d1** | 2.182 | 1.144, 4.163 | **0.018** |
| **Vasoactive therapy @ d1** | 0.960 | 0.573, 1.607 | 0.875 |
| **Constant** | 0.230 |  | 0.017 |
| N = 764; Goodness of fit (Hosmer & Lemeshow) Chi^2^ 11.963, df 8, p = 0.153, Percentage correctly predicted 88.1 %, AUC-ROC 0.735, 95%CI: 0.687, 0.784, p<0.001 | | | |

# Results: Additional table 5b: Risk factors for developing AKI based on creatinine criteria only (multivariable logistic regression analysis)

| **Variable** | **OR** | **95% CI** | **P** |
| --- | --- | --- | --- |
| **Age** | 1.009 | 0.994, 1.025 | 0.242 |
| **BMI>30** | 1.561 | 1.095, 2.226 | **0.014** |
| **CKD** | 2.274 | 1.497, 3.452 | **<0.001** |
| **Hypertension** | 1.147 | 0.805, 1.634 | 0.447 |
| **Diabetes** | 1.398 | 0.967, 2.020 | 0.075 |
| **APACHE II** | 1.054 | 1.026, 1.083 | **<0.001** |
| **IMV on d1** | 2.542 | 1.747, 3.698 | **<0.001** |
| **Vasoactive therapy on d1** | 1.009 | 0.709, 1.436 | 0.960 |
| **Constant** | 0.047 |  | **<0.001** |
| N = 764; Goodness of fit (Hosmer & Lemeshow) Chi^2^ 4.748, df 8, p = 0.784, Percentage correctly predicted 71.1%, AUC-ROC 0.731, 95%CI: 0.694, 0.768, p<0.001 | | | |

# Results: Additional table 5c: Risk factors for developing AKI in the cohort of patients with known baseline serum creatinine (multivariable logistic regression analysis)

| **Variable** | **OR** | **95% CI** | **P** |
| --- | --- | --- | --- |
| **Age** | 1.042 | 1.021, 1.063 | **<0.001** |
| **BMI>30** | 3.225 | 1.813, 5.737 | **<0.001** |
| **CKD** | 1.275 | 0.622, 2.614 | 0.507 |
| **Hypertension** | 0.759 | 0.449, 1.283 | 0.303 |
| **Diabetes** | 1.151 | 0.643, 2.060 | 0.635 |
| **APACHE II** | 1.033 | 0.990, 1.078 | **0.139** |
| **IMV on d1** | 2.077 | 1.065, 4.049 | **0.032** |
| **Vasoactive therapy on d1** | 1.042 | 0.615, 1.764 | 0.879 |
| **Constant** | 0.198 |  | 0.012 |
| N = 723; Goodness of fit (Hosmer & Lemeshow) Chi^2^ 10.149, df 8, p = 0.255, Percentage correctly predicted 88.0%, AUC-ROC 0.732, 95%CI: 0.681, 0.782, p<0.001 | | | |

Legend: BMI = body mass index, CKD = chronic kidney disease, IMV@d1 = invasive mechanical ventilation at day 1 of ICU admission, AUC-ROC = area under the curve of the receiver operating characteristic curve, CI = confidence interval

# Results: Additional table 6a: Variables associated with ICU mortality

| Variable | ICU alive | ICU death | P |
| --- | --- | --- | --- |
| AKI | 773 (81.2%) | 322 (96.4%) | <0.001 |
| AKI full KDIGO |  |  | <0.001 |
| No AKI | 179 (18.8%) | 12 (3.6%) |  |
| AKI stage 1 | 124 (13.0%) | 31 (9.3%) |  |
| AKI stage 2 | 476 (50.0%) | 134 (40.1%) |  |
| AKI stage 3 | 173 (18.2%) | 157 (47.0%) |  |
| AKI-sCr |  |  | <0.001 |
| No AKI | 662 (69.5%) | 108 (32.3%) |  |
| AKI stage 1 | 172 (18.1%) | 103 (30.8%) |  |
| AKI stage 2 | 42 (4.4%) | 29 (8.7%) |  |
| AKI stage 3 | 76 (8.0%) | 94 (28.1%) |  |
| AKI-UO |  |  | <0.001 |
| No AKI | 204 (21.5%) | 33 (9.9%) |  |
| AKI stage 1 | 114 (12.0%) | 21 (6.3%) |  |
| AKI stage 2 | 482 (50.8%) | 148 (44.4%) |  |
| AKI stage 3 | 149 (15.7%) | 131 (39.3%) |  |
| AKI stage 2 or 3 | 649 (68.2%) | 291 (87.1%) | <0.001 |
| KRT | 55 (5.8%) | 71 (21.3%) | <0.001 |
| Gender male | 654 (68.7%) | 225 (67.4%) | 0.652 |
| Age | 66 (17) | 75 (13) | <0.001 |
| Age >65y (n =1285) | 490 (51.5%) | 270 (80.8%) | <0.001 |
| Body weight (kg) | 83 (21.6) | 81 (19.5) | 0.037 |
| BMI | 28.0 (7.3) | 27.8 (6.4) | 0.388 |
| Obese (BMI>30) (n =1262) | 345 (36.9%) | 108 (33.0%) | 0.209 |
| LOS hosp | 6 (6) | 4 (6) | <0.001 |
| LOS ICU | 8 (6.9) | 6 (6) | <0.001 |
| Hypertension (n = 1042) | 348 (43.6%) | 144 (59.3%) | <0.001 |
| CV disease (n = 948) | 269 (38.1%) | 122 (50.4%) | <0.001 |
| COPD/Asthma (n= 949) | 99 (14.0%) | 46 (18.9%) | 0.067 |
| Diabetes (n = 955) | 178 (24.3%) | 67 (30.0%) | 0.086 |
| Liver disease (n =948) | 14 (2.0%) | 4 (1.7%) | 0.745 |
| Malignancy (n = 947) | 72 (10.2%) | 40 (16.5%) | 0.009 |
| Immunosuppressed (n=948) | 63 (8.9%) | 32 (13.2%) | 0.055 |
| Creatinine baseline (mg/dL) | 0.82 (0.38) | 0.93 (0.48) | <0.001 |
| eGFR baseline | 86.6 (28.5) | 72.7 (36.9) | <0.001 |
| CKD (n = 1285) | 150 (15.8%) | 102 (30.5%) | <0.001 |
| ACEI/ARBs (n = 727) | 171 (32.0%) | 71 (36.8%) | 0.229 |
| Corticosteroids (all) (n= 1043) | 577 (72.2%) | 203 (83.2%) | <0.001 |
| Immunosuppressive therapy (n = 947) | 40 (5.7%) | 16 (6.6%) | 0.594 |
| APACHE II | 15 (9) | 19 (13) | <0.001 |
| SAPS II | 39 (19) | 48 (34) | <0.001 |
| SOFA | 4 (6) | 8 (9) | 0.002 |
| Lymphocytes (n = 813) | 775 (790) | 585 (1888) | 0.022 |
| Lymphocytes below 1000 | 371 (62.7%) | 147 (66.5%) | 0.310 |
| Lymphocytes categories |  |  | 0.001 |
| Lymphocytes<500 | 156 (26.4%) | 87 (39.4%) |  |
| Lymph500-1000 | 215 (36.3%) | 60 (27.1%) |  |
| Lymph >1000 | 221 (37.3%) | 74 (33.5%) |  |
| Ferritin (µg/L) | 1236 (1503) | 1331 (1286) | 0.087 |
| CRP (mg/dl) | 152.3 (160.6) | 144.4 (127.7) | 0.112 |
| D-dimers ((ng/mL)) | 1140 (2334) | 1479 (5768) | <0.001 |
| PaO2/FiO2 | 93 (60) | 77 (45) | <0.001 |
| FiO2 (%) | 75 (41) | 80 (37) | 0.001 |
| MAP (mmHg) | 74 (24) | 67 (21) | <0.001 |
| Hydrochloroquine  (n = 1285) | 271 (28.54%) | 62 (18.6%) | <0.001 |
| Remdesivir  (n = 1285) | 146 (15.3%) | 47 (14.1%) | 0.591 |
| Anti-IL1 or anti-IL6 (n = 1315) | 43 (4.5%) | 11 (3.3%) | 0.337 |
| Convalescent COVID-19 plasma  (n = 1214) | 12 (1.3%) | 3 (1.0%) | 0.621 |
| GM-CSF (Leukine®) (n = 221) | 3 (1.8%) | 0 | 1.000 |
| NSAIDs (n = 1285) | 38 (4.0%) | 10 (3.0%) | 0.413 |
| Aminoglycoside  (n = 1041) | 47 (5.9%) | 36 (14.9%) | <0.001 |
| Vancomycine  (n = 1285) | 137 (14.4%) | 78 (23.4%) | <0.001 |
| HFO till d21  (n = 923) | 391 (56.3%) | 121 (53.1%) | 0.401 |
| NIV (n = 952) | 57 (7.7%) | 34 (15.9%) | <0.001 |
| IMV d1 (n = 962) | 251 (34.2%) | 100 (43.9%) | 0.008 |
| IMV till d21(n = 870) | 390 (59.8%) | 170 (78.0%) | <0.001 |
| Prone | 48 (7.4%) | 15 (7.5%) | 0.970 |
| ECMO | 18 (2.8%) | 14 (6.9%) | 0.009 |
| Blood type AB/A  (n = 782) | 321 (61.6%) | 164 (62.8%) | 0.740 |
| Vasoactive therapy d1 | 263 (27.6%) | 124 (37.1%) | 0.001 |
| Vasoactive therapy till d21 | 457 (48.0%) | 191 (57.2%) | 0.004 |
| LOS ICU (days) | 13 (21) | 13 (17) | 0.011 |
| IMV duration (days) | 6 (14) | 10 (15) | <0.001 |
| Vasoact therapy duration (days) | 2 (7) | 7 (10) | <0.001 |
| LOS hospital (days) | 18 (38.8) | 14 (22.3) | 0.006 |

Legend: AKI = acute kidney injury, ICU = intensive care unit, KDIGO = Kidney Disease: Improving Global Outcomes, AKI-sCr = AKI based on creatinine criteria only, AKI-UO = AKI based on urine output criteria only, KRT = kidney replacement therapy, y = year, BMI = body mass index, LOS = length of stay, COVID = Corona virus disease, hosp = hospital, CV = cardiovascular, COPD = chronic obstructive pulmonary disease, Immuno = immune compromised, eGFR = estimated glomerular filtration rate, CKD = chronic kidney disease, ACEI = angiotensin converting enzyme inhibitors, ARBs = angiotensin receptor blockers, CRP = c-reactive protein, PaO2= arterial oxygen pressure, FiO2 = fraction of inspired oxygen, MAP = mean arterial pressure, mmHg = millimeters of mercury, IL = interleukin, GM-CSF = granulocyte-macrophage colony-stimulating factor, NSAIDs= non-steroidal anti-inflammatory drugs, HFO = high flow oxygen, d = day, NIV = non-invasive ventilation, IMV = invasive mechanical ventilation, ECMO = extracorporeal membrane oxygenation, LOS = length of stay

# Results: Additional table 6b: Variables associated with ICU mortality (logistic regression analysis) – unadjusted

| Variable | OR | 95% CI | P |
| --- | --- | --- | --- |
| No AKI | 1 |  |  |
| AKI stage 1 | 3.729 | 1.843, 7.544 | <0.001 |
| AKI stage 2 | 4.199 | 2.270, 7.768 | <0.001 |
| AKI stage 3 | 13.537 | 7.260, 25.243 | <0.001 |

Legend: AKI = acute kidney injury, OR = odds ratio, CI = confidence interval

# Results: Additional table 6c: Variables associated with ICU mortality (multivariable logistic regression analysis) – adjusted odds ratios

| Variable | OR | 95% CI | P |
| --- | --- | --- | --- |
| AKI |  |  | <0.001 |
| AKI stage 1 | 3.179 | 1.054, 9.583 | 0.040 |
| AKI stage 2 | 3.024 | 1.139, 8.028 | 0.026 |
| AKI stage 3 | 12.039 | 4.418, 32.802 | <0.001 |
| Age | 1.080 | 1.056, 1.1050 | <0.001 |
| Hypertension | 0.842 | 0.557, 1.273 | 0.415 |
| COPD | 1.197 | 0.733, 1.953 | 0.473 |
| Diabetes | 0.849 | 0.549, 1.313 | 0.461 |
| Malignancy | 1.120 | 0.648, 1.937 | 0.684 |
| Immunosuppression | 2.339 | 1.227, 4.458 | 0.010 |
| eGFR baseline | 1.001 | 0.991, 1.010 | 0.878 |
| APACHE II | 1.029 | 0.999, 1.061 | 0.061 |
| PaO2/FiO2 D1 | 0.995 | 0.991, 0.999 | 0.008 |
| Vasoactive therapy d1 | 1.245 | 0.833, 1.861 | 0.285 |
| Constant | 0.000 |  | <0.001 |
| N = 743, Goodness of fit (Hosmer & Lemeshow) Chi^2^ 6.661, df 8, p = 0.573; Percentage correctly predicted 78.5%; AUC-ROC 0.806, 95% CI: 0.771, 0.841, p<0.001 | | | |

Legend: AKI = acute kidney injury, OR = odds ratio, CI = confidence interval, COPD = chronic obstructive pulmonary disease, eGFR = estimated glomerular filtration rate, PaO2 = arterial oxygen pressure, FiO2 = fraction of inspired oxygen, d = day, AUC-ROC = area under the curve of the receiver operating characteristic curve

# Results: Additional table 6d: Variables associated with ICU mortality (multivariable logistic regression analysis) –odds ratios for adjusted models in cohorts defined by the KDIGO AKI definition, the variants based on AKI-sCR or AKI-UO, and in the cohort with known baseline serum creatinine

| Variable | OR | 95% CI | P |
| --- | --- | --- | --- |
| AKI in the whole cohort | | | |
| AKI full KDIGO | | | |
| AKI |  |  | <0.001 |
| AKI stage 1 | 3.179 | 1.054, 9.583 | 0.040 |
| AKI stage 2 | 3.024 | 1.139, 8.028 | 0.026 |
| AKI stage 3 | 12.039 | 4.418, 32.802 | <0.001 |
| AKI-sCr |  |  |  |
| AKI |  |  | <0.001 |
| AKI stage 1 | 3.319 | 2.026, 5.345 | <0.001 |
| AKI stage 2 | 4.417 | 1.921, 10.621 | <0.001 |
| AKI stage 3 | 9.943 | 5.696, 17.355 | <0.001 |
| AKI-UO |  |  |  |
| AKI |  |  | <0.001 |
| AKI stage 1 | 1.474 | 0.597, 3.641 | 0.401 |
| AKI stage 2 | 1.784 | 0.891, 3.570 | 0.102 |
| AKI stage 3 | 5.636 | 2.676, 11.868 | <0.001 |
|  |  |  |  |
| AKI defined on true baseline sCr only | | | |
| AKI full KDIGO |  |  |  |
| AKI |  |  | <0.001 |
| AKI stage 1 | 3.040 | 0.994, 9.292 | 0.051 |
| AKI stage 2 | 2.774 | 1.037, 7.418 | 0.042 |
| AKI stage 3 | 11.788 | 4.284, 32.438 | <0.001 |
| AKI-sCr |  |  |  |
| AKI |  |  | <0.001 |
| AKI stage 1 | 3.286 | 1.958, 5.513 | <0.001 |
| AKI stage 2 | 5.729 | 2.226, 14.743 | <0.001 |
| AKI stage 3 | 10.414 | 5.810, 18.664 | <0.001 |
| AKI-UO |  |  |  |
| AKI |  |  | <0.001 |
| AKI stage 1 | 1.295 | 0.506, 3.311 | 0.590 |
| AKI stage 2 | 1.607 | 0.795, 3.426 | 0.186 |
| AKI stage 3 | 5.330 | 2.499, 11.368 | <0.001 |

Legend: AKI = acute kidney injury, AKI-sCr = AKI based on creatinine criteria only, AKI-UO = AKI based on urine output criteria only, OR = odds ratio, CI = confidence interval

# Results: Additional table 6e: Variables associated with ICU mortality (multivariable logistic regression analysis) in patients with AKI-UO who do not fulfill AKI-sCr criteria – adjusted odds ratios

| Variable | OR | 95% CI | P |
| --- | --- | --- | --- |
| AKI-UO |  |  | 0.084 |
| AKI-UO stage 1 | 2.482 | 0.727, 8.470 | 0.147 |
| AKI-UO stage 2 | 2.055 | 0.738, 5.724 | 0.168 |
| AKI-UO stage 3 | 4.836 | 1.393, 16.792 | **0.013** |
| Age | 1.074 | 1.038, 1.110 | **<0.001** |
| Hypertension | 0.887 | 0.481, 1.636 | 0.702 |
| COPD | 1.291 | 0.648, 2.570 | 0.467 |
| Diabetes | 0.862 | 0.431, 1.724 | 0.675 |
| Malignancy | 1.380 | 0.629, 2.570 | 0.422 |
| Immunodepression | 2.934 | 1.167, 7.377 | **0.022** |
| eGFR | 0.994 | 0.979, 1.010 | 0.482 |
| APACHE II | 1.031 | 0.982, 1.082 | 0.226 |
| PaO2/FiO2 D1 | 0.990 | 0.984, 0.997 | **0.007** |
| Vasoactive therapy d1 | 1.336 | 0.736, 2.428 | 0.341 |
| Constant | 0.001 |  | <0.001 |
| N = 488, Goodness of fit (Hosmer & Lemeshow) Chi^2^ 4.997, df 8, p = 0.758;  Percentage correctly predicted 85.9% | | | |

Legend: AKI = acute kidney injury, AKI-UO = AKI based on urine output criteria only, OR = odds ratio, CI = confidence interval, COPD = chronic obstructive pulmonary disease, eGFR = estimated glomerular filtration rate, PaO2 = arterial oxygen pressure, FiO2 = fraction of inspired oxygen, d = day

# Results: Additional table 7: Comparison of patient characteristics in the first versus the second COVID-19 wave

|  | **First wave** | **Second Wave** | **p** |
| --- | --- | --- | --- |
| Age (years) | 67 (57, 76) | 70 (60, 77) | **0.002** |
| Sex (male) | 69.9% | 67.4% | 0.345 |
| BMI  (mg/kg²) | 27.8 (25.0, 31.6) | 27.8 (25.0, 32.2) | 0.943 |
| BMI >=30 | 35.8% | 35.9% | 0.976 |
| Baseline sCr  (mg/dl) | 0.90 (0.69, 1.09) | 0.82 (0.67, 1.07) | **0.045** |
| eGFR (ml/kg/1.73m²) | 82 (66, 98) | 85 (68, 98) | 0.543 |
| CKD | 20.6% | 19.0% | 0.477 |
| Hypertension | 45.6% | 48.3% | 0.387 |
| Diabetes | 25.9% | 25.5% | 0.907 |
| COPD/Asthma | 13.4% | 16.6% | 0.175 |
| Malignancy | 10.5% | 12.7% | 0.299 |
| APACHE II | 12 (16, 22) | 17 (13, 22) | 0.528 |
| SAPS 2 | 40 (33, 50.8) | 38 (30, 47) | **0.002** |
| SOFA | 5 (3, 10) | 5 (2, 9) | **0.033** |
| Acute Kidney Injury variants | | | |
| AKI | 86.3% | 84.5% | 0.374 |
| AKI-sCr | 42.6% | 38.1% | 0.103 |
| AKI-UO | 83.7% | 80.1% | 0.105 |
| KRT | 11.9% | 8.3% | **0.035** |
| Rapid Reversal | 8.1% | 10.7% | 0.116 |
| AKD | 88.7% | 86.7% | 0.301 |
|  |  |  |  |
| Corticosteroids home | 4.2% | 8.5% | **0.011** |
| Corticosteroids hospital | 44.4% | 95.2% | **<0.001** |
| Hydroxychloroquine | 67.4% | 0% | **<0.001** |
| Remdesivir | 1.6% | 23.4% | **<0.001** |
| Anti IL-1/-6 therapy | 5.5% | 3.4% | 0.076 |
| Convalescent plasma | 5.5% | 3.4% | 0.076 |
| NSAIDs | 2.6% | 4.4% | 0.099 |
| Aminoglycoside | 6.7% | 4.4% | 0.214 |
| Vancomycin | 16.4% | 16.9% | 0.799 |
|  |  |  |  |
| Lymphocytes <1000 | 52.4% | 70.6% | **<0.001** |
|  |  |  |  |
| HFO | 46.2% | 62.0% | **<0.001** |
| NIV | 4.1% | 6.9% | 0.072 |
| IMV day 1 | 40.2% | 33.9% | **0.048** |
| Vasoactive therapy day 1 | 38.4% | 24.9% | **<0.001** |

Legend: AKI = acute kidney injury, ICU = intensive care unit, KDIGO = Kidney Disease: Improving Global Outcomes, AKI-sCr = AKI based on creatinine criteria only, AKI-UO = AKI based on urine output criteria only, KRT = kidney replacement therapy, y = year, BMI = body mass index, LOS = length of stay, COVID = Corona virus disease, hosp = hospital, CV = cardiovascular, COPD = chronic obstructive pulmonary disease, Immuno = immune compromised, eGFR = estimated glomerular filtration rate, CKD = chronic kidney disease, ACEI = angiotensin converting enzyme inhibitors, ARBs = angiotensin receptor blockers, CRP = c-reactive protein, PaO2= arterial oxygen pressure, FiO2 = fraction of inspired oxygen, MAP = mean arterial pressure, mmHg = millimeters of mercury, IL = interleukin, GM-CSF = granulocyte-macrophage colony-stimulating factor, NSAIDs= non-steroidal anti-inflammatory drugs, HFO = high flow oxygen, d = day, NIV = non-invasive ventilation, IMV = invasive mechanical ventilation, ECMO = extracorporeal membrane oxygenation, LOS = length of stay

# Results: Additional table 8: ICU mortality according to AKI Stage: comparison between first and second COVID-19 wave

|  | **ICU mortality**  **First wave (n = 495)** | **ICU mortality**  **Second Wave (n = 791)** | P |
| --- | --- | --- | --- |
| **All** | 21.2% | 29.0% | **0.002** |
| **No AKI** | 1.5% | 8.9% | 0.059 |
| **AKI** **1** | 24.4% | 32.6% | **0.003** |
| **AKI 2** | 12.5% | 24.2% | 0.079 |
| **AKI 3** | 14.5% | 26.3% | **<0.001** |
|  |  |  |  |

Legend: AKI = acute kidney injury, AKI-sCr = AKI based on creatinine criteria only, AKI-UO = AKI based on urine output criteria only, ICU = intensive care unit

# Acknowledgements

Participants (alphabetic order)

AZ St Jan, Bruges

Camille Bourgeois, MD

Marc Bourgeois, MD

Joke Denolf

Bram Dewulf, MD

Ziekenhuis Oost-Limburg, Genk

Willem Boer, MD

Ben Goethuys

Tom Fivez, MD, PhD

Dieter Messotten, MD, PhD

Noella Pierlet

Ghent University Hospital

Veerle Brams, RN

Pieter Depuydt, MD, PhD

Anoushka Desmeytere, RN

Eric Hoste, MD, PhD

Hannah Schaubroeck, MD

Wim Vandenberghe, MD

Jolien Van Hecke, RN

Daisy Vermeiren, RN

Jessa Ziekenhuis, Hasselt

Ina Callebaut

Jasperina Dubois, MD

Laurien Geebelen

Björn Stessel, MD, PhD

UZ Leuven

Greet De Vlieger, MD, PhD

Jan Gunst, MD, PhD

Greet Hermans, MD, PhD

Philippe Meersseman, MD, PhD

Alexander Wilmer, MD, PhD

Joost Wauters, MD, PhD

Pieter Wouters

AZ Delta, Roeselaere

Alexander Dumoulin, MD

Piet Lormans, MD

AZ Turnhout, Turnhout

Eva Boonen, MD, PhD

Marc Vanhoof, MD

# References

1. KDIGO Group. KDIGO Clinical Practice Guideline for Acute Kidney Injury. Kidney Int Suppl. 2012;2:1-138.

2. Chawla LS, Bellomo R, Bihorac A, Goldstein SL, Siew ED, Bagshaw SM, et al. Acute kidney disease and renal recovery: consensus report of the Acute Disease Quality Initiative (ADQI) 16 Workgroup. Nature reviews Nephrology. 2017;13(4):241-57.

3. Nadim MK, Forni LG, Mehta RL, Connor MJ, Jr., Liu KD, Ostermann M, et al. COVID-19-associated acute kidney injury: consensus report of the 25th Acute Disease Quality Initiative (ADQI) Workgroup. Nature reviews Nephrology. 2020;16(12):747-64.

4. Levey AS, Bosch JP, Lewis JB, Greene T, Rogers N, Roth D. A more accurate method to estimate glomerular filtration rate from serum creatinine: a new prediction equation. Modification of Diet in Renal Disease Study Group. Annals of internal medicine. 1999;130(6):461-70.

5. Hoste EA, Clermont G, Kersten A, Venkataraman R, Angus DC, De Bacquer D, et al. RIFLE criteria for acute kidney injury are associated with hospital mortality in critically ill patients: a cohort analysis. Critical care (London, England). 2006;10(3):R73.

6. Lameire NH, Levin A, Kellum JA, Cheung M, Jadoul M, Winkelmayer WC, et al. Harmonizing acute and chronic kidney disease definition and classification: report of a Kidney Disease: Improving Global Outcomes (KDIGO) Consensus Conference. Kidney international. 2021;100(3):516-26.

7. Kellum JA, Sileanu FE, Bihorac A, Hoste EA, Chawla LS. Recovery after Acute Kidney Injury. American journal of respiratory and critical care medicine. 2017;195(6):784-91.

8. Le Gall JR, Lemeshow S, Saulnier F. A new Simplified Acute Physiology Score (SAPS II) based on a European/North American multicenter study. JAMA. 1993;270(24):2957-63.

9. Vincent JL, Moreno R, Takala J, Willatts S, De Mendonca A, Bruining H, et al. The SOFA (Sepsis-related Organ Failure Assessment) score to describe organ dysfunction/failure. On behalf of the Working Group on Sepsis-Related Problems of the European Society of Intensive Care Medicine. Intensive care medicine. 1996;22(7):707-10.

10. Knaus WA, Draper EA, Wagner DP, Zimmerman JE. APACHE II: a severity of disease classification system. Critical care medicine. 1985;13(10):818-29.

11. Donnelly JP, Chen SC, Kauffman CA, Steinbach WJ, Baddley JW, Verweij PE, et al. Revision and Update of the Consensus Definitions of Invasive Fungal Disease From the European Organization for Research and Treatment of Cancer and the Mycoses Study Group Education and Research Consortium. Clinical infectious diseases : an official publication of the Infectious Diseases Society of America. 2020;71(6):1367-76.

12. World Health Organisation. Available from: <https://www.who.int/news-room/fact-sheets/detail/obesity-and-overweight>.

13. COVID-19 Epidemiologische situatie [Internet]. Sciensano. 2021. Available from: <https://covid-19.sciensano.be/nl/covid-19-epidemiologische-situatie>.
